# Discrete bHLH transcription factors play functionally overlapping roles in pigmentation patterning in flowers of *Antirrhinum majus*

**DOI:** 10.1111/nph.17142

**Published:** 2021-01-12

**Authors:** Nick W. Albert, Eugenio Butelli, Sarah M.A. Moss, Paolo Piazza, Chethi N. Waite, Kathy E. Schwinn, Kevin M. Davies, Cathie Martin

**Affiliations:** ^1^ Plant & Food Research Food Industry Science Centre Fitzherbert Science Centre Batchelar Road Palmerston North 4474 New Zealand; ^2^ John Innes Centre Norwich Research Park NR4 7UH UK; ^3^ Oxford Genomics Centre University of Oxford Roosevelt Drive Oxford, OX3 7BN UK

**Keywords:** anthocyanin, *Antirrhinum majus*, floral pigment patterning, MBW complex, transcriptional regulation

## Abstract

- Floral pigmentation patterning is important for pollinator attraction as well as aesthetic appeal. Patterning of anthocyanin accumulation is frequently associated with variation in activity of the Myb, bHLH and WDR transcription factor complex (MBW) that regulates anthocyanin biosynthesis.
- Investigation of two classic mutants in *Antirrhinum majus*, *mutabilis* and *incolorata I*, showed they affect a gene encoding a bHLH protein belonging to subclade bHLH‐2. The previously characterised gene, *Delila*, which encodes a bHLH‐1 protein, has a bicoloured mutant phenotype, with residual lobe‐specific pigmentation conferred by Incolorata I.
- Both Incolorata I and Delila induce expression of the anthocyanin biosynthetic gene *DFR*. Rosea 1 (Myb) and WDR1 proteins compete for interaction with Delila, but interact positively to promote Incolorata I activity. Delila positively regulates *Incolorata I* and *WDR1* expression. Hierarchical regulation can explain the bicoloured patterning of *delila* mutants, through effects on both regulatory gene expression and the activity of promoters of biosynthetic genes like *DFR* that mediate MBW regulation.
- bHLH‐1 and bHLH‐2 proteins contribute to establishing patterns of pigment distribution in *A. majus* flowers in two ways: through functional redundancy in regulating anthocyanin biosynthetic gene expression, and through differences between the proteins in their ability to regulate genes encoding transcription factors.

## Introduction

The diversity of coloration and patterning of animal‐pollinated flowers is truly remarkable. Complex patterns serve as nectar guides, attracting pollinators and directing them towards pollen and nectar rewards. Most of our understanding of pigmentation patterning comes from the study of regulation of anthocyanin production, which provides red/purple/blue colours to plants. Patterns of anthocyanin production across petals arise primarily through transcriptional regulation of the anthocyanin biosynthetic genes (Schwinn *et al*., 2006), which are regulated directly by a transcriptional activation complex comprised of R2R3‐Myb, bHLH and WDR proteins; the MBW complex (Baudry *et al*., 2004; Koes *et al*., 2005; Ramsey & Glover, 2005; Gonzalez *et al*., 2008). The R2R3‐Myb proteins are particularly important for pattern formation because they are encoded by gene families with members that are expressed differentially in response to a variety of developmental and environmental cues (Schwinn *et al*., 2006; Hoballah *et al*., 2007; Albert *et al*., 2011; Yuan *et al*., 2014; Bombarely *et al*., 2016; Esfeld *et al*., 2018). The bHLH transcription factors are also encoded by small gene families and they too have the potential to contribute to pigment patterning (Dellaporta *et al*., 1988; Chandler *et al*., 1989; Ludwig *et al*., 1989; Goodrich *et al*., 1992; Hellens *et al*., 2010).

MBW complexes also regulate proanthocyanidin biosynthesis (Baudry *et al*., 2004; Bogs *et al*., 2007; Terrier *et al*., 2009; Liu *et al*., 2014), vacuolar hyperacidification (Quattrocchio *et al*., 2006; Butelli *et al*., 2019), and trichome and root hair development in *Brassica* species (Payne *et al*., 2000; Zhao *et al*., 2008) with each process controlled by a specific subgroup of Myb proteins (Zhang *et al*., 2003; Xu *et al*., 2014; Zhang & Hulskamp, 2019). Most plants have at least two bHLH transcription factors that operate within MBW complexes, belonging to two distinct clades within subgroup IIIf of plant bHLH proteins (Heim *et al*., 2003; Feller *et al*., 2011). These are described as bHLH‐1 (represented by *Zm*R/*Zm*Lc, *At*GL3, *At*EGL3, *At*MYC1, *Ph*JAF13, *Am*Del) and bHLH‐2 (represented by *Zm*In, *At*TT8, *Ph*AN1, *Cs*Noemi) (Albert *et al*., 2014). In many groups of plants the activity of bHLH‐2 genes is essential for anthocyanin biosynthesis (Spelt *et al*., 2000, 2002; Park *et al*., 2004; Hellens *et al*., 2010; Butelli *et al*., 2019; Strazzer *et al*., 2019), In other plants, including Arabidopsis (Gonzalez *et al*., 2008; Feyissa *et al*., 2009) and *Antirrhinum* (Goodrich *et al*., 1992), the bHLH‐1 proteins act also in controlling anthocyanin biosynthesis directly (Zhang *et al*., 2003; Zhang & Hulskamp, 2019). bHLH‐2 proteins appear to be essential for controlling proanthocyanidin biosynthesis in Arabidopsis (Nesi *et al*., 2000), petunia (Spelt *et al*., 2002), pea (Hellens *et al*., 2010), morning glory (Park *et al*., 2007), medicago (Li *et al*., 2016) and citrus (Butelli *et al*., 2019), and vacuolar acidification in petunia (Spelt *et al*., 2002) and citrus (Butelli *et al*., 2019; Strazzer *et al*., 2019). It is not yet clear whether bHLH‐1 proteins that regulate anthocyanin biosynthesis also contribute to regulating these other pathways. The existence of genes encoding both clades of bHLH proteins in monocots and dicots suggests that there has been selection to maintain two types of bHLH protein which may work by distinct mechanisms or have distinct functions (Pesch *et al*., 2015; Zhang *et al*., 2019).

### *Antirrhinum majus* – a classic model for understanding floral pigmentation

In bicoloured flowers, colour patterning differs between individual petals or zones within petals and is common in zygomorphic flowers such as legumes (banner, wings, keel), iris (standards, falls), violas, *Aquilegia* (blades, spurs) and orchids (tepals, labellum). However, suitable models with bicoloured floral patterns that are also amenable to genetic analyses have not been analysed extensively. Wild‐type *Antirrhinum majus* has bilaterally symmetrical flowers consisting of self‐coloured fused petals in the corolla tube which separate into two hind petals, two lateral petals and a single ventral petal comprising the corolla lobes. However, mutants of the bHLH‐1 gene *Delila* have bicoloured flowers (Goodrich *et al*., 1992), providing a model genetic system for investigating bicolour patterning. Three R2R3Myb proteins, Rosea 1 (Ros 1), Rosea 2 and Venosa, of which Ros 1 has the strongest activity, control flower pigmentation in *A. majus* (Schwinn *et al*., 2006). Here we report the identification and functional characterisation of *Incolorata I*, a gene encoding a bHLH‐2 protein in *A. majus*, and relate this to bicoloured patterning of its flowers.

## Materials and Methods

### Plant material

The *del* mutant, line JI:8, is closely related to the wild‐type line, JI:7. The *incolorata I* mutant, line Jl:569, was obtained from Professor Linnert, Frei University of Berlin in 1984 and outcrossed to JI:7. Segregation in the F_2_ allowed the selection of ivory *inc I *:* del* homozygotes. The *mutabilis* mutant was identified from several individuals segregating in an F_2_ population from a cross between a stock line carrying *decipiens* and JI:7 (Stubbe, 1966). The origins of *del^rec^* (JI:602; JI22) and *del^23^* (JI:23) have been described by Goodrich *et al*. (1992) and Martin *et al*. () and lines JI:32, JI:33, JI:56, JI:520, and JI:522 have been described by Fincham & Harrison, (1967), Coen *et al*., (1986) and Almeida *et al*., (1989).

### Construction of a cDNA library from RNA from lobes of del flowers

RNA was extracted from red petal lobes from a *del* line (Jl:8). cDNA was synthesised from polyA^+^ RNA. *Eco*Rl linkers were ligated to the cDNA ends and cloned into the *Eco*Rl site of λgt10. The resultant library contained *c*. 6 × 10^6^ PFU

### Screening of the cDNA library

Approximately 5 × 10^5^ PFU were screened using a ^32^P‐labelled fragment from a cDNA clone of *An1* (Spelt *et al*., 2000) amplified from petals of line V26. Three positive plaques were identified. A 2.4 kb *Kpn*I fragment from the largest cDNA insert was subcloned into the *Kpn*I site of pBluescript (Stratagene) and named pJAM1494.

### RNA preparation for RNA gel blots

RNA was extracted from petals and RNA gel blots were run as described by Martin *et al*. (1985). Probes for biosynthetic genes were prepared as described in Martin *et al*. (1991) and Schwinn *et al*. (2006).

### Isolation of genomic clones

A library of genomic DNA from JI:522 was prepared in λEMBL4 (Martin *et al*., 1991) and screened with pJAM1494. DNA inserts from positive plaques were subcloned as *Eco*RI fragments into pBluescript. DNA was also isolated from *inc I^1 ^*: *del* and *inc I^2 ^*: *del* lines digested with *Eco*RI, size‐fractionated and cloned into λNM1149. Inserts were screened with pJAM1494 and the positive *Eco*RI inserts were subcloned into pGEM‐T (Promega) and sequenced.

Amplification of *inc I*, *delila* and *Delila‐like* alleles from genomic DNA was performed with iProof polymerase (Bio‐Rad) and gene‐specific primers (Supporting Information Table S1). Sequences have been submitted to GenBank with the following accession numbers: *del^23^*, genomic DNA, MW027119: *inc I^1^*, genomic DNA, MW027120; *Incolorata I*, cDNA, MW027121; *WDR1*, cDNA, MW027122.

### Phylogenetic tree

Deduced amino acid sequences were aligned using Muscle (Edgar, 2004) to generate a maximum likelihood phylogenetic tree using PhyML (Guindon *et al*., 2010) with 1000 bootstrap replicates using Geneious (10.0.9) software. Amino acid sequence alignments are shown in Dataset S1.

### Biolistic transformation of *inc I* petals

Complementation assays of *inc I^1 ^*: *del* petals were performed by biolistic transformation of petals (Albert *et al*., 2015). GFP‐ER was used as an internal control for identifying transformed cells (Haselhoff *et al*., 1997).

### Isolation of *AmWDR1*

*WDR1* was isolated by 3′ Rapid Amplification of cDNA Ends (RACE) PCR (Frohman *et al*., 1988) from line JI:75 using degenerate oligonucleotides K112 and K113 for first and second amplification rounds, respectively. The 5′ end of the *WDR1* gene was isolated using a Universal Genome Walker kit (Clontech) and gene‐specific primers K127 and K128 for the first and second amplification rounds, respectively. The full‐length *WDR1* cDNA was amplified from RNA from petals using primers K133 and K134 and cloned into pJAM1502. For all primers see Table S1.

### RNA isolation and qRT‐PCR

Whole petals of wild‐type (JI:522), *rosea^dorsea^* and *incolorata I* flowers, or dissected wild‐type (JI:522) and *delila* (JI:8) petals (tubes and lobes) were sampled. Total RNA was extracted using Plant RNeasy Isolation kits (Qiagen). For qRT‐PCR analysis, cDNA was prepared from 1 µg total RNA using IScript™ gDNA clear cDNA synthesis kits (Bio‐Rad), and diluted 20‐fold for analysis. qRT‐PCR using SsoAdvanced™ Universal SYBR^®^ Green Supermix (Bio‐Rad), gene‐specific primers (Table S1) were normalised to the geometric mean of *Cyclophilin* and *EF1α* (Albert *et al*., 2014). Means ± SEM of three biological replicates were calculated. Two‐tailed Student’s *t*‐tests between wild‐type and the corresponding mutant samples were performed to determine significant differences. Data were transformed (log10) to account for unequal variance.

Full‐length transcripts of *Delila* and *Delila‐like* were amplified with gene‐specific primers (Table S1) and 2GRobust polymerase (Kapa Biosciences), from cDNA synthesised using Superscript II™ reverse transcriptase (Life technologies) and oligo (dT)_12–18_.

### Transient dual luciferase assays in *Nicotiana benthamiana*

The ability of different transcription factors to activate the promoter of the *Pallida* gene encoding dihydroflavonol‐4‐reductase (*DFR*) was assessed by combining transient expression via agroinfiltration of *N. benthamiana* leaves with a quantitative dual luciferase reporter system.

Effector plasmids encoding transcription factors were obtained by cloning coding sequences first into the pDONR207 entry clone (Thermo Fisher Scientific/Invitrogen) and subsequently transferred to the destination vectors pJAM1502 (Ros, Inc1, WDR1) or pMDC32 (Del) using Gateway cloning technology (Thermo Fisher Scientific/Invitrogen). Both vectors are Gateway‐compatible binary plasmids that allow strong, constitutive expression of the genes of interest driven by double CaMV 35S promoters (Curtis & Grossniklaus, 2003; Luo *et al*., 2007).

The reporter constructs p2532, p2534 and p2536 were obtained by PCR amplification of the *DFR* promoter from different *A. majus* accessions followed by cloning into the pGreen II 0800‐LUC vector as *Kpn*I*–Nco*I fragments to control the expression of the firefly‐derived luciferase reporter gene. This vector also contains a *Renilla* luciferase gene under the control of a CaMV 35S promoter as an internal control to normalise the values of the experimental reporter gene for variations caused by transfection efficiency (Hellens *et al*., 2005). The reporter constructs containing deletions in the *DFR* promoter (p2551, p2557, p2565 and p2571) were obtained using the Q5 Site‐Directed Mutagenesis Kit (New England Biolabs) using plasmid p2532 as a template and primers listed in Table S1.

Effector and reporter plasmids were transformed into *Agrobacterium tumefaciens* strain GV3101. Reporter plasmids were co‐transformed with the helper plasmid pSoup (Hellens *et al*., 2005). Liquid cultures were grown overnight with selection (kanamycin 50 mg l^−1^, rifampicin 25 mg l^−1^) and harvested by centrifugation. Cells were washed and resuspended in 10 mM MgCl_2_, 10 mM MES pH 5.6, 200 μM acetosyringone to A_600_ = 0.2. *Agrobacterium* suspensions were infiltrated into the abaxial surface of expanded leaves of 3‐wk‐old *N. benthamiana* plants. For each combination, five injected areas were treated as biological replicates. Agroinfiltrated leaves were harvested after 3 d and luciferase activity was measured immediately using the Dual‐Glo Luciferase Assay System kit (Promega). Leaf discs of 4 mm in diameter were collected in 1.5 ml white transparent tubes containing 100 μl of PBS. A volume of 75 μl of luciferase assay reagent was added and firefly luminescence was measured on a Glomax 20/20 single tube luminometer (Promega) after 10 min. *Renilla* luminescence was measured on the same instrument 10 min after the addition to the same sample of 75 μl of Stop & Glo reagent. Results were expressed as the ratio of firefly to *Renilla* luciferase activity (Luc/Ren).

## Results

### Classic mutants *incolorata I* and *mutabilis* are allelic and affect anthocyanin biosynthesis

**Fig. 1:**
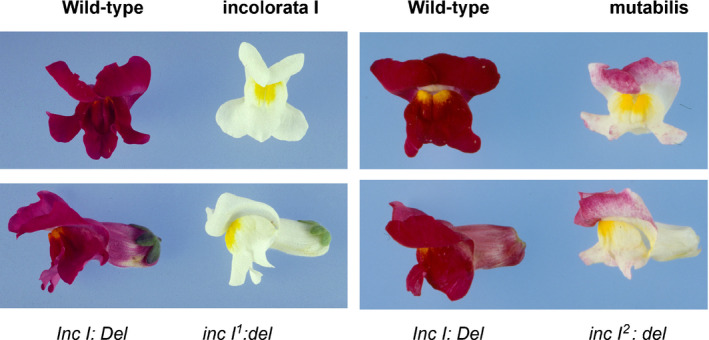
Phenotypes of *incolorata I* and *mutabilis* mutants compared with wild‐type full red flowers (JI:7) of *Antirrhinum majus*. Phenotypes are written above the images and their genotypes are shown below the images. The *del* knockout mutant has a colourless tube but fully pigmented lobes.

The classic *delila* mutant of *Antirrhinum majus* has bicoloured flowers with red lobes, pigmented with anthocyanin, and an ivory‐coloured tube (Wheldale, 1907; Baur, 1910). *Del* encodes a bHLH transcription factor that regulates anthocyanin pigmentation in both tubes and lobes (Goodrich *et al*., 1992). However, knockout mutants of *del* have fully pigmented lobes and consequently the bicolour pattern in *del* mutants has been predicted to arise from the activity of a second, unknown protein, active in the petal lobes (Goodrich *et al*., 1992; Schwinn *et al*., 2006). Two mutants affect lobe pigmentation: *incolorata I* (*inc I*) and *mutabilis* (*mut*) (Stubbe, 1966; Linnert, 1972). The *inc I* mutant was originally identified by its ivory flowers and complete absence of anthocyanins in vegetative tissues (Fig. 1) (Stubbe, 1966; Linnert, 1972). The *inc I* mutant line was obtained from the IPK Gatersleben collection of *Antirrhinum* germplasm in 1984 and outcrossed to JI:7 (a full red, self‐coloured lin*e* (*Inc I*/*Inc I *:* Del*/*Del*)). Segregation in the F_2_ showed 9/56 plants segregating for *del* in addition to full red (43/56: *Inc I*/*Inc I* and *Inc I*/*inc I*) and ivory *inc I *:* del* homozygotes (4/56). This suggested that the original *inc I* mutant line was homozygous for *del*, which was confirmed by crossing a reselected *inc I* line to JI:8 (*del*/*del*). All F1 plants lacked tube pigmentation (*del*/*del*) but had full red lobes, confirming that *inc I* was a recessive mutation, visible only in combination with homozygous *del* alleles (Fig. 1).

The *mutabilis* mutation was described originally by a series of alleles in the IPK Gatersleben collection, linked (2 cM) to *Del* (Stubbe, 1966). While these original mutants have been lost from the collection, we identified an individual with the *mutabilis* phenotype from an F_2_ population from a cross between a stock line carrying *decipiens* (Stubbe, 1966) and wild‐type (JI:7). The rediscovered *mutabilis* mutant has pale pink blushing/speckling on the petal lobes (Fig. 1), but completely lacks tube pigmentation because it is homozygous for *del*. The speckling of the lobes is much more evident in plants grown in the field than in those grown in the glasshouse (Fig. S1) with strong pigmentation on the abaxial epidermis of the lobes except where tissue has been shaded before unfolding, suggesting that this allele is sensitive to light. An F2 population from a cross between the *mutabilis* mutant (*mut:del*) and a full red wild‐type line (JI:7 *Mut:Del*), segregated for flower colour patterning. Phenotypes fell into three categories: ivory tube with pale, speckled lobes (*mut:del* parental phenotype; 56 plants), full red tube and lobes (wild‐type parental phenotype; 200 plants) and ivory tube with red lobes (*del* phenotype; 8 plants). Recombinants with a red tube and pale, speckled lobes were not observed, indicating that *Del* activity is epistatic to *Mut* in regulating pigmentation. Further attempts to replace *del* with *Del* alleles in the *mut* mutant line by outcrossing proved unsuccessful, confirming that the *mut* phenotype, like that of *inc I*, is observed only in the presence of nonfunctional *del* alleles.

Crosses between *inc I* and *mut* did not complement the pigmentation phenotype, showing the mutations to be allelic (Fig. S2). The gene was named *Incolorata I*, and the *incolorata I* mutant allele was named *inc I^1^* (Stubbe, 1966; Linnert, 1972). The *mutabilis* mutant carries a weak allele of *inc I*, designated *inc I^2^*.

The redundancy of *Inc I* with *Del* in regulating anthocyanin biosynthesis suggested that the genes encode similar proteins. *Del* encodes a bHLH transcription factor (Goodrich *et al*., 1992), belonging to the R/JAF13 bHLH‐1 clade of subgroup IIIf (Heim *et al*., 2003). In other species, a second class of bHLH proteins (TT8/AN1 bHLH‐2 clade) regulates anthocyanin and proanthocyanidin biosynthesis (Nesi *et al*., 2000; Spelt *et al*., 2002). We screened cDNA libraries generated from lobe tissue with a *del* background with a cDNA probe of the *AN1* gene from petunia line V26. Multiple cDNA clones corresponding to a new bHLH sequence were identified: the longest cDNA insert was subcloned as a 2.4 kb *Kpn*I insert in pBluescript (pJAM1494). Sequencing confirmed this contained a full‐length cDNA. The wild‐type *Incolorata I* gene was identified in a 24 kb genomic DNA insertion in λEMBL4 from line JI:522. Fragments of the gene were also cloned from *inc I^1^* genomic DNA and sequenced. A 4 bp duplication (CATG) was identified within exon three of the *inc I^1^* mutant line, resulting in a frame‐shift, confirming this gene to be *Incolorata I*. We were unable to identify any sequence variants in genomic DNA that could account for the weak phenotype of the *inc I^2^* allele but RNA gel blots from wild‐type and *inc I^2^* mutant flowers (Fig. S3) revealed an absence of *Inc I* transcript in the mutant, suggesting that the *inc I^2^* mutant phenotype results from considerably reduced levels of *Inc I* expression.

**Fig. 2:**
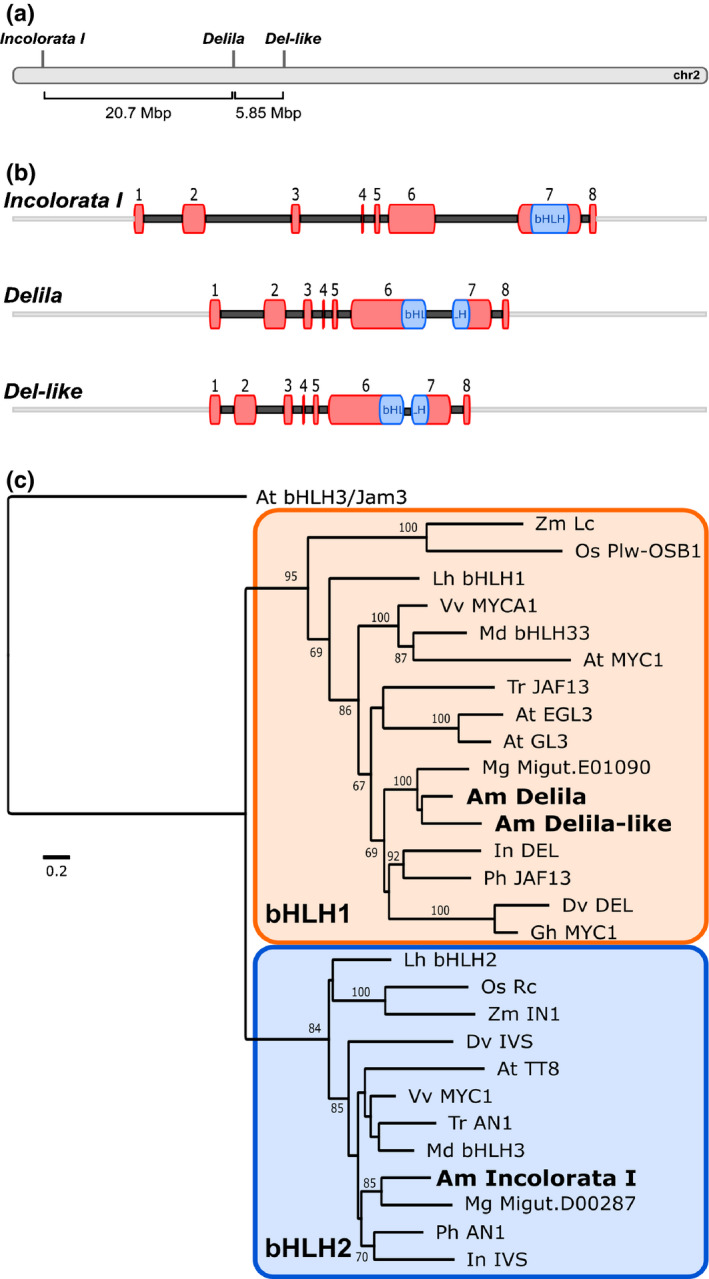
Molecular and phylogenetic analysis of Incolorata I, Delila and Del‐like bHLH transcription factors. (a) Location of *Incolorata I*, *Delila* and *Delila‐like* genes on Chromosome 2 of *Antirrhinum majus*. (b) Structure of *Delila*, *Delila‐like* and *Incolorata I* bHLH genes. Exons are numbered and the bHLH domain is indicated in blue. (c) Maximum likelihood phylogenetic tree of subgroup IIIf bHLH proteins, with *At*bHLH3 (subgroup IIId) included as an outgroup (Heim *et al*., 2003). 1000 bootstrap replicates, ≥60% support indicated. Gene IDs/accession numbers of sequences used: *A. majus* Am Delila (AAA32663.1; *Am*02g33340); Delila‐like (Am02g28470); Incolorata I (*Am*02g53780) (*A. majus* genome, v.3); *Arabidopsis thaliana* At bHLH3/JAM3 (NP_193376); EGL3 (OAP12509); GL3 (NP_680372); TT8 (Q9FT81); *At*MYC1 (NP_001154194); *Dahlia variabilis* DvDel (BAJ33516); IVS (BAJ33515); *Gossypium hirsutum* Gh: MYC1 (CAA07615); *Ipomoea nil* InDel (XP_019171149); IVS (XP_019197480); *Lilium hybrida* LhbHLH1 (BAE20057); bHLH2 (BAE20058); *Malus domestica* MdbHLH3 (ADL36597); bHLH33 (ABB84474); *Mimulus gattatus* (*Erythranthe guttata*) MgMigut.E01090.1; Migut.D00287.1; *Oryza sativa* OsPlw‐OsB1 (BAB64301); Rc (ADK36625); *Petunia hybrida* Ph AN1 (AAG25928); JAF13 (AAC39455); *Trifolium repens* TrTrAN1 (AIT76559) *Tr*JAF13 (AIT76563); *Vitis vinifera* Vv MYC1 (NM_001281253); MYCA1 (NP_001267954); *Zea mays* Zm Lc (AAA33504); In1 (AAB03841).

Previous genetic data suggested that *Mut*/*Inc I* and *Del* were closely linked, 2 cM apart (Stubbe, 1966). From our crosses of *inc I^2^ *:* del* × *Inc I *:* Del* a total of 264 F_2_ plants had 64 *del*/*del* individuals of which 56 had speckled/ivory flowers (*inc I^2^* homozygotes) and 8 (*del*/*del*) individuals had red lobes and so carried at least one *Inc l* allele. Assuming all the latter to be heterozygous *Inc I *:* inc I^2^*, we calculated there to have been 8 recombination events among 128 gametes giving a recombination fraction (r) of 8/128 = 0.0625 (± 0.0214) and the distance between *Inc I* and *Del*, (d) *c*. 6.7 (± 2.19) cM, meaning *Inc I* and *Del* lie about 6.7 cM apart. In the high quality genome sequence for *A. majus* (Li *et al*., 2019) *Incolorata I* (Am02g53780) and *Delila* (Am02g33340) are both located on chromosome 2, 20.7 Mb apart (Fig. 2a).

Lying 5.85 Mb beyond *Del* on chromosome 2 is a sequence encoding a third bHLH protein, very similar to Del, which we named Delila‐like (Am02g28470) (Fig. 2a,c). The *Delila‐like* gene encodes a bHLH transcription factor in the bHLH‐1 clade, and its intron/exon structure (established by amplification of a cDNA), was characteristic of bHLH‐1 genes (Fig. 2b).

Phylogenetic analysis of the predicted amino acid sequences of Incolorata I, Delila and Delila‐like together with other bHLH transcription factors that regulate flavonoid biosynthesis confirmed that they represent the two bHLH clades within subgroup IIIf (Fig. 2c). The sequence similarity and the conserved exon/intron structure between *Inc I* and genes such as *AN1* (petunia) and *TT8* (Arabidopsis), with the bHLH domain encoded entirely in exon 7, placed Inc I firmly within the bHLH‐2 clade.

### Confirmation of functional redundancy of Del, Del‐like and Inc I in controlling anthocyanin biosynthesis

**Fig. 3:**
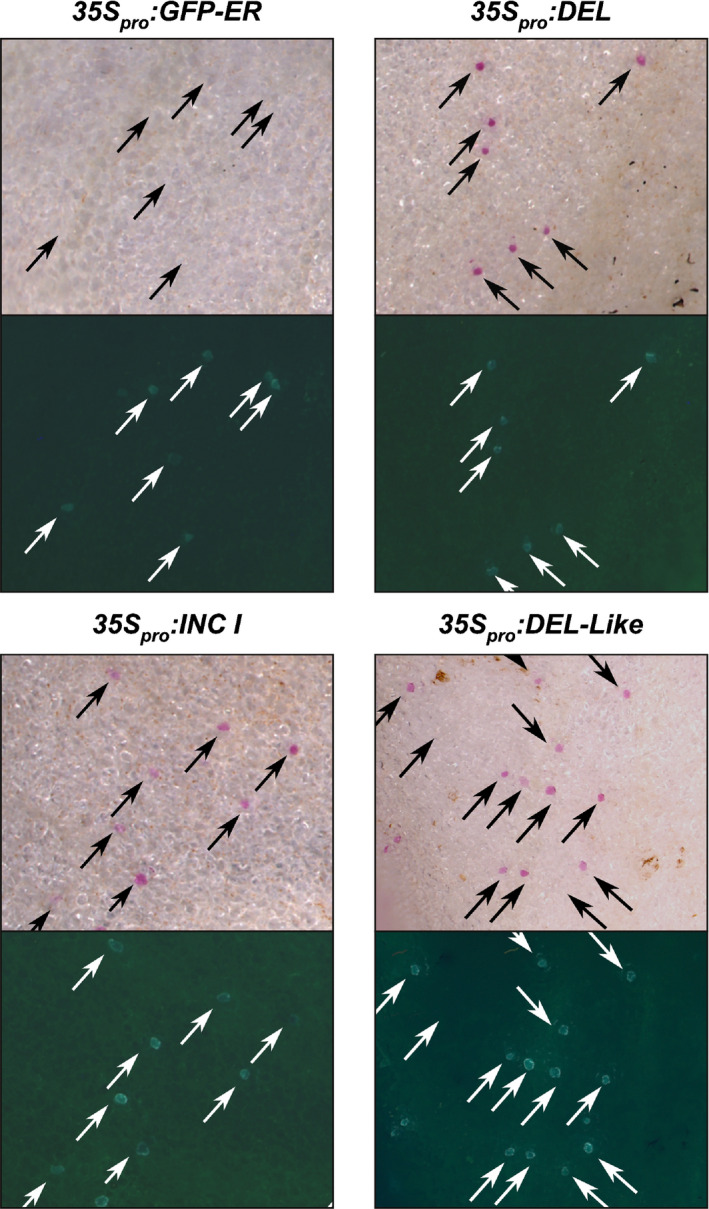
Complementation of *incolorata I^1^* with bHLH transcription factors. *Inc I^1^* petals of *A. majus* were biolistically transformed with *35S_pro _*: *Delila*, *35S_pro _*: *Inc I or 35S_pro _*: *Delila‐like*, co‐transformed with *GFP‐ER* to identify transformed cells (indicated with arrows). Red anthocyanin accumulation was visible in cells transformed with *Delila*, *Delila‐like* or *Inc I*, but was not detected with the GFP‐ER control.

We confirmed the functional redundancy of Inc I, Del and Del‐like in inducing anthocyanin biosynthesis by bombarding petal lobes of the *inc I:del* double mutant line with plasmids carrying each cDNA driven by the CaMV 35S promoter. All three genes were able to restore anthocyanin production to single cells of the petal lobes, by contrast to the CaMV 35S: GFP control (Fig. 3).

### Genetic evidence that Del acts redundantly with Inc I in inducing anthocyanin pigmentation

**Fig. 4:**
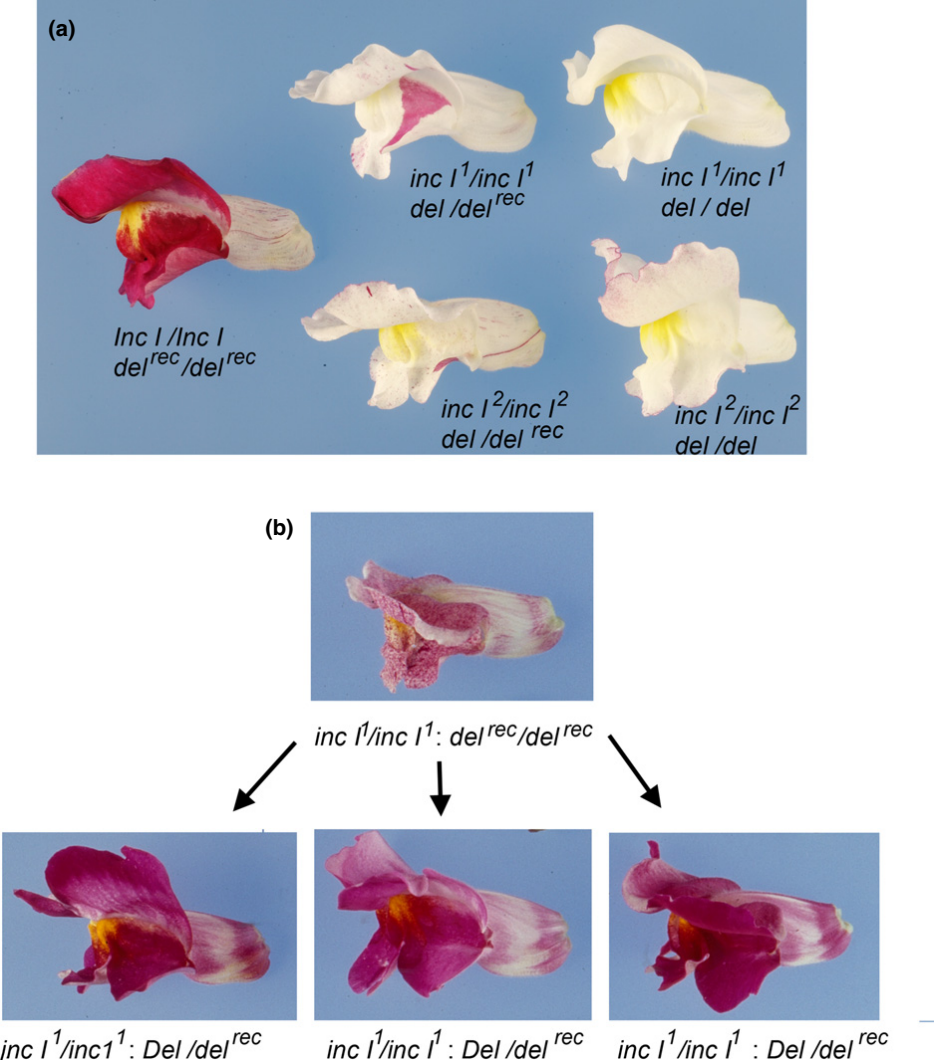
*Inc I* acts redundantly with *Del* in controlling pigmentation in petal lobes of *A. majus*. (a) Revertant sectors of *Del* arising from transposon excision from *del^rec^* complement fully loss of *inc I* function in lobes of *inc I *:* del^rec^* flowers segregating in F_2_ populations of *inc I^1 ^*:*^ ^del* × *Inc I *:* del^rec^* and *inc I^2 ^*:*^ ^del* × *Inc I *:* del^rec^* crosses. (b) Independent full red germinal revertants from an *inc I^1^*/ *inc I^1 ^*:*^ ^del^rec^*/*del^rec^* parental line have full red lobes and tubes in F_3_ plants.

Crosses were performed between *incolorata I* (*inc I^1^*), *mutabilis* (*inc I^2^*) and an unstable *delila^recurrens^* mutant (*del^rec^*). JI:602 *del^rec^* flowers have red petal lobes, but the petal tubes are ivory with red sectors, resulting from the excision of a transposable element within the *Del* gene, that restores *Del* activity (Goodrich *et al*., 1992). *Del* revertant sectors were able to complement both *inc I^1^* and *inc I ^2^* mutant backgrounds, restoring pigmentation in lobes (Fig. 4a). Revertant *Del* sectors were observed that extended throughout the tube and lobes, confirming *Del* to be active in both tissues and to mask the phenotype of *inc I* mutations (Figs 4,S4). Individuals were recovered in the F_3_ generation where the transposable element had excised from the *Del* locus in the germline of an *incI*/*incI *:* del^rec^*/*del* parental plant. These plants had full red flowers with coloured lobes and tubes (Fig. 4b).

**Fig. 5:**
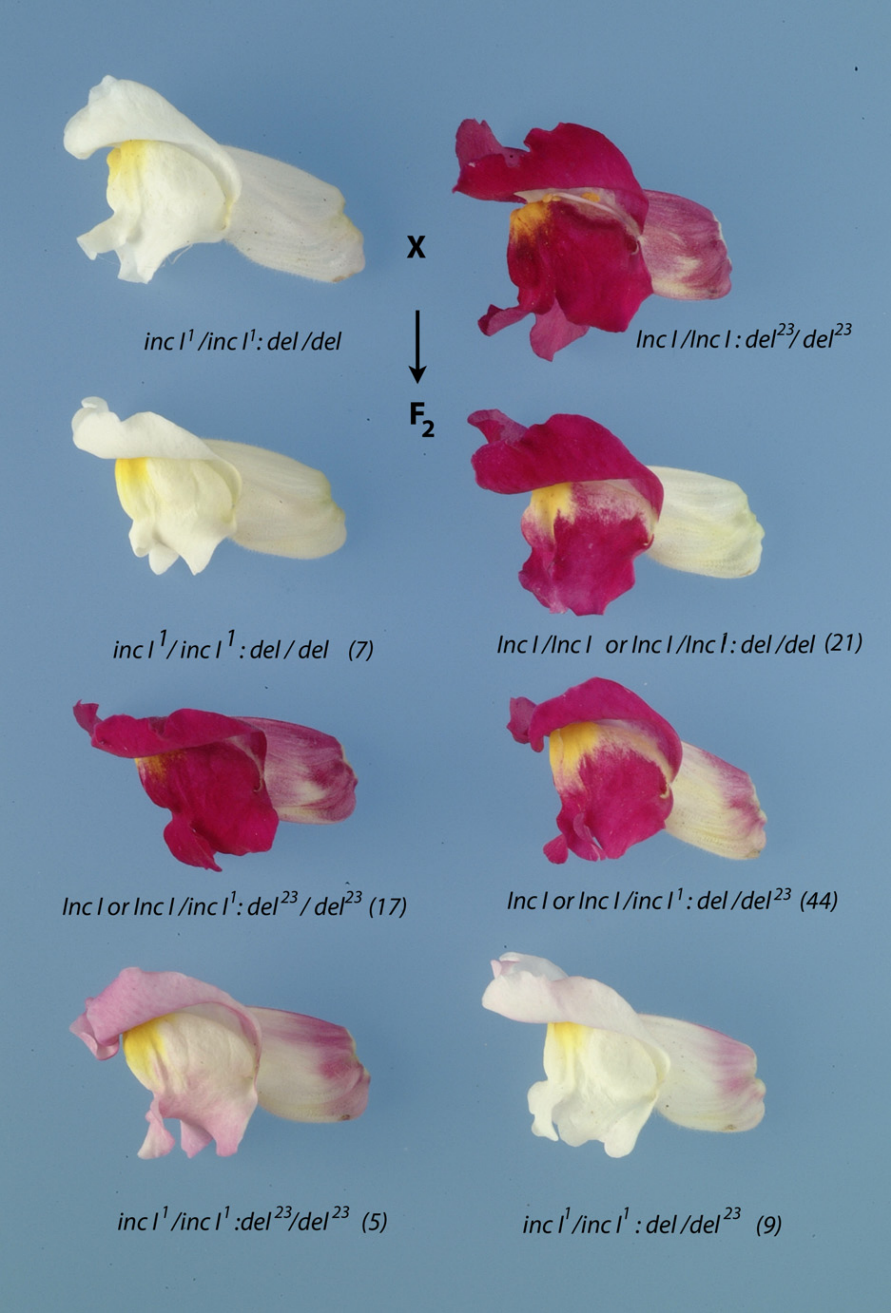
Phenotypes of individual plants segregating in the *A. majus* F_2_ population of *Inc I *:* del* ^23^ x *inc I^1 ^*:*^ ^del* cross. Top row: Floral phenotypes of the parental lines *Inc I *:* del* ^23^ and *inc I^1 ^*:*^ ^del*. Second, third and fourth rows: Floral phenotypes that segregated in the F_2_ generation. The genotypes conferring these phenotypes are shown below each flower together with the number of individuals with that phenotype from a total F_2_ population of 96 individual plants (shown in parentheses).

The functional redundancy between *Inc I* and *Del* was investigated further by a cross between the stock line JI:23 (Martin *et al*., ) and *inc I^1^*. JI23 carries a weak allele of *Delila* (*del^23^*) that results in slightly reduced anthocyanin pigmentation in the tube, particularly the upper region of the tube (Fig. S5). The F_2_ population from JI:23 *Inc I*/*Inc I *:* del^23^*/*del^23^* × *inc I^1^*/*inc I^1^* : *del*/*del* segregated for flower colour in 96 plants scored, with six distinct phenotypes. In addition to the parental *inc I^1^* : *del* and *Inc I *:* del^23^* phenotypes, four recombinant phenotypes were observed: 21 plants were *del* (ivory tube) of which seven were *inc I^1^ *:*^ ^del*; 61 plants were *Inc I*/*Inc I* or *Inc I*/*inc I^1^* with *del^23^*, nine plants were *inc I^1^*/*inc I^1^; del*/*del^23^* with a flush of pigmentation on the lobes and 5 plants were *inc I^1^*/*inc I^1^* : *del^23^*/*del^23^* with a dark flush on the lobes (Fig. 5). The phenotypic classes and their frequencies were consistent with segregation between *del^23^* and *del* such that the phenotype of *del^23^* with a flush of pigmentation on the lobes (9/96) was due to the *inc I^1^*/*inc I^1^* : *del^23^*/*del* genotype and the phenotype of *del^23^* with a dark flush on lobes (5/96) was due to the *inc I^1^*/*inc I^1^* : *del^23^*/*del^23^* genotype. These genotypes were confirmed in the F_3_ where the *del^23^* with a dark flush on lobes (*inc I^1^*/*inc I ^1 ^*:*^ ^del^23^*/*del^23^*) bred true, and *del^23^* with a flush of pigmentation on the lobes segregated three *del^23^* with a dark flush on lobes (*inc I^1^*/*inc I^1 ^*:*^ ^del^23^*/*del^23^*): nine *del^23^* with a flush of pigmentation on the lobes (*inc I^1^*/*inc I^1 ^*:*^ ^del^23^*/*del*): three ivory flowers (*inc I^1^*/*inc I^1 ^*:*^ ^del*/*del*), from a family of 15 plants. These data demonstrated that *Del* acts within both the tube and lobes to regulate anthocyanin pigmentation, acting redundantly with *Inc I* in the lobes, but predominating in the tube of the flowers.

Interestingly by comparing the flowers of *Inc I*/*Inc I *:* del^23^*/*del^23^* with those of *inc I^1^*/*inc I^1 ^*:*^ ^del^23^*/*del^23^*it was apparent that *inc I* activity affected anthocyanin accumulation in the tubes of the flowers as well as in the lobes, although its contribution to anthocyanin accumulation is substantially greater in the lobes than in the tubes (Fig. 5). This was confirmed by comparing the colour of *Inc I*/*Inc I *:* del^23^*/*del* flowers to *inc I^1^*/*inc I^1 ^*:*^ ^del^23^*/*del* flowers (Fig. 5). By contrast, although Del operates in both the lobes and the tubes, its contribution is substantially greater in tubes than in lobes as shown by comparing the phenotypes of *inc I^1^*/*inc I^1 ^*: *del^23^*/*del* and *inc I^1^*/*inc I^1 ^*: *del^23^*/*del^23^* (Fig. 5).

To confirm that *del^23^* was indeed a weak allele of *Del*, cDNA and genomic DNA were amplified from flowers of JI:23 (Fig. S6). The genomic sequence of *Del* in JI:23 (*del^23^*) had 29 SNPs within the coding sequence (14 were nonsynonymous) compared with the reference genome (Goodrich *et al*., 1992; Li *et al*., 2019), additional SNPs (79) and small insertions into introns, including one near a splice acceptor site within intron five (Fig. S6b) that resembled an intron acceptor site. Sequences of 8/11 clones of the *Del* cDNA from this line were mis‐spliced into the insertion, resulting in mRNA sequences that encoded an insertion of an extra amino acid within a conserved region of the predicted Del protein, one transcript spliced correctly, one retained two introns and one had an entire portion of the cDNA missing (Fig. S6b). We concluded that this insertion causes mis‐splicing of the *Del* mRNA in a significant proportion of the transcripts in JI:23 flowers, confirming that *del^23^* was a weak *del* allele.

### Identification of target genes of Inc I in anthocyanin biosynthesis

**Fig. 6:**
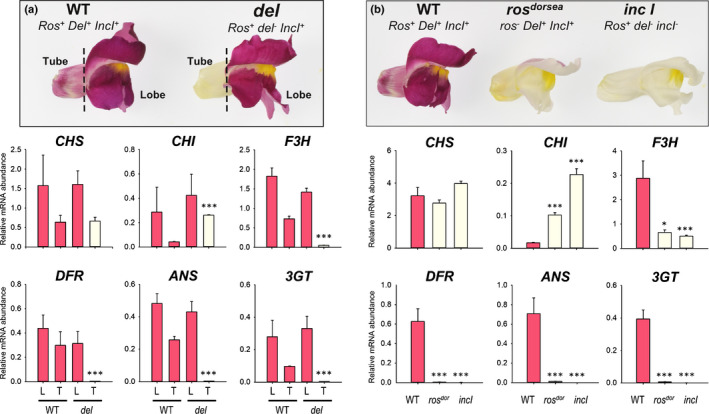
Anthocyanin biosynthesis genes are downregulated in *incolorata I* ^1^ petals of *A. majus*. (a) Wild‐type (WT) and *delila* mutant flowers were separated into tube and lobes for analysis of *CHS*, *CHI*, *F3H*, *DFR*, *ANS* and *3GT* transcript abundance by qRT‐PCR in tube (T) and lobe (L) tissue of WT and *delila* mutant petals. (b) Petals from whole flowers of WT, *ros^dorsea^* and *inc I^1 ^*: *del* were analysed for *CHS*, *CHI*, *F3H*, *DFR*, *ANS* and *3GT* transcript levels by qRT‐PCR. Bar colour indicates pigmentation status. Means ± SEM, *n* = 3 biological replicates. Means that are significantly different (Student’s *t‐*test) from WT are indicated (*, *P* < 0.05; **, *P* < 0.01; ***, *P* < 0.001).

To determine the target genes regulated by Inc I, transcript analyses of biosynthetic genes were conducted in dissected tubes and lobes of *del* or wild‐type flowers (Fig. 6a) or whole petals of wild‐type, *rosea^dorsea^* (a mutant of *Rosea 1* which encodes an R2R3Myb transcription factor) and *inc I^1 ^*: *del* flowers (Fig. 6b). Transcripts of the genes encoding DFR, anthocyanidin synthase (ANS) and flavonoid: 3‐glycosyltransferase (3GT) were at trace amounts in *inc I^1 ^*: *del* flowers and the transcript levels of flavanone 3‐hydroxylase (F3H) were substantially reduced. Transcripts of the genes encoding chalcone synthase (CHS) and chalcone isomerase (CHI), were not reduced (Fig. 6a). Similarly, transcript abundance for *F3H*, *DFR*, *ANS* and *3GT* were severely reduced in whole petals of *rosea^dorsea^*, and *inc I^1^ *:*^ ^del* (Fig. 6b). These data were confirmed by RNA gel blots of the *inc I^2 ^*: *del* double mutant, *mutabilis* (Fig. S7) establishing that mutation of *inc I* causes down regulation of expression of the genes encoding F3H, DFR, ANS and 3GT (Fig. 6a; Martin *et al*. 1991), but not CHS or CHI. These effects were very similar to those of mutations in *Del* (Martin *et al*., 1991; Jackson *et al*., 1992), suggesting that the two transcription factors share the same target genes in anthocyanin biosynthesis.

### Model of MBW regulation of *Pallida* (DFR)

Some time ago, Almeida *et al*. (1989) proposed a model for bicoloured patterning of *delila* mutants based on analysis of mutations in the promoter of the *Pallida* (*pal*) gene encoding DFR (Coen *et al*., 1986; Almeida *et al*., 1989), which proposed that Del and a lobe‐specific transcription factor bound to the same upstream region of the *DFR* promoter (Box C: Fig. S8) in tubes and lobes respectively. At the time, none of the regulatory genes had been identified but, with these regulatory genes now to hand, we tested this model using some of the same *DFR* promoter mutations. We expressed *Del*, *Inc I*, *Ros 1* and a cDNA encoding a WD repeat protein from *A. majus* homologous to An11 in petunia (WDR1) under the control of the CaMV35S promoter in *Nicotiana benthamiana*, and tested the different *Antirrhinum* *DFR* promoter sequences using the dual luciferase assay to measure promoter responsiveness.

**Fig. 7:**
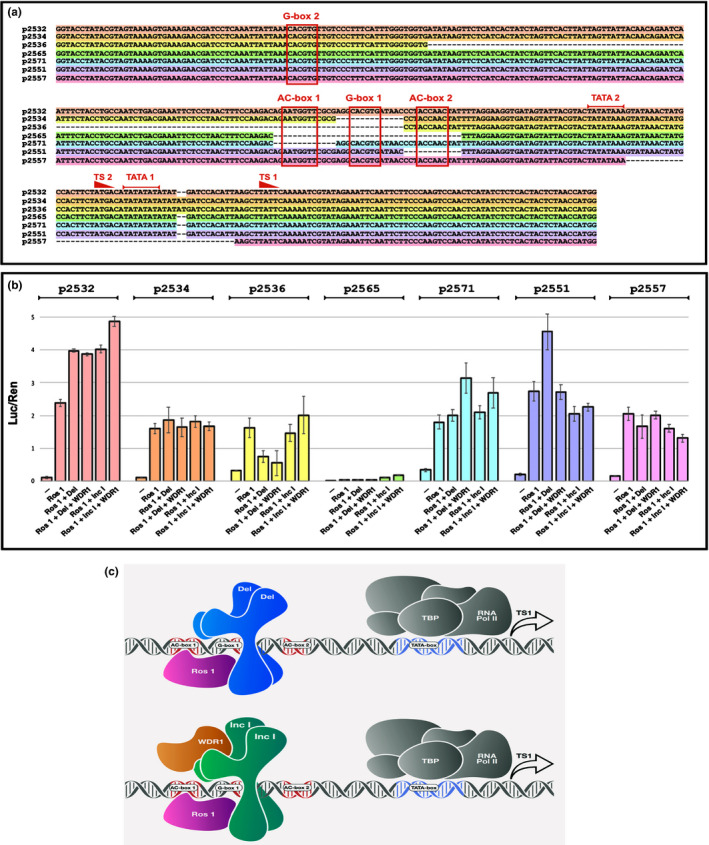
Assessment of responsiveness of the *DFR* promoter from *A. majus* to components of the MBW complex from *A. majus* using dual luciferase assays in *N. benthamiana*. (a) Structure of the wild‐type promoter of the *DFR* gene from *A. majus* (p2532) showing the gene proximal transcriptional start site (TS1) and associated TATA box (TATA 1) and the gene distal transcriptional start site (TS2) and its associated TATA box (TATA 2) (Coen *et al*., 1986).G‐box 1 is shown flanked on its gene distal side by AC‐box 1 and on its gene proximal side by AC‐box 2. It should be noted that AC‐box 1 reads 5′ to 3′ on the lower DNA strand whereas AC‐box 2 reads 5′ to 3′ on the upper strand. The location of a second G‐box (G‐box 2) is also shown. Below the sequence of the WT promoter (p2532; highlighted in pink) the deletions assayed using the dual luciferase assay are shown, coloured in accordance with the bars detailing the effects of these deletions on luciferase reporter activity shown in panel b). (b) Activation of luciferase by MBW components on wild‐type *DFR* promoter (pink) and promoters carrying deletions of the key upstream activating sequences (UAS) detailed in panel (a). All values relate luciferase to Renilla luminescence and are the results from at least five independent assays. Error bars show SEMs. (c) Model illustrating the predominant binding of MBW components to the wild‐type *DFR* promoter from *A. majus*. The bHLH proteins Del and Inc I bind to G‐box 1 probably as a dimer. The N‐termini of Del and Inc I interact with the surface exposed region of R3 of the Myb DNA binding domain of Ros1 carrying the bHLH interaction signature motif (Zimmermann *et al*., 2004). TBP = TATA Binding Protein For Inc I the additional binding by WDR1 is necessary for full activity.

The wild‐type *DFR* promoter has two starts of transcription (TS), one 66 bp upstream of the initiating ATG (TS1) and the second (TS2), 99 bp upstream of the initiating ATG (Coen *et al*., 1986). TS1 is the stronger initiation site in *A. majus* (Coen *et al*., 1986; Robbins *et al*., 1989). TS1 is preceded by a TATA box (TATA‐1: −86 to −93) and TS2 is preceded by TATA‐2 (−120 to −127; Fig. 7a). There was significant induction of luciferase driven by the wild‐type *DFR* promoter (p2532) by Ros 1 alone. This was possibly due to binding of Ros 1 to an AC‐box which lies between −184 and −190 nucleotides upstream of the initiating ATG codon of the *DFR* gene (AC‐box 1; Fig. 7a) or to a second AC‐box (AC‐box 2) lying between −157 and −162 bp upstream of the start of translation of the *DFR* gene. This transcriptional response to Ros 1 alone may be a feature specific to tobacco. AC‐box 1 is likely to be a Myb binding site, because its deletion following transposon excision reduced *DFR* gene expression and anthocyanin production to very low levels in the tube of the flower (Almeida *et al*., 1989). Just eight bp gene proximal to the AC‐box 1 box is G‐box 1 (CACGTG: −171 to −176), a recognised binding site for bHLH transcription factors (Fig. 7a; equivalent to Box B in Almeida *et al*., 1989; Goodrich *et al*., 1992). Displacement of these sequences (AC‐box 1 box and G‐box 1) from the downstream TATA boxes/transcriptional start sites causes complete loss of *DFR* expression in *A. majus* as shown by the insertion of Tam 3 at −169 bp upstream of the initiating ATG in the *DFR* promoter (Fig. S8; Carpenter *et al*., 1987).

There was substantial induction of luciferase activity when Del was included together with Ros 1 on the wild‐type *DFR* promoter (p2532), but no further enhancement was observed when WDR1 was also included (Fig. 7b). This suggested that Ros 1 and Del act together to induce *DFR* expression and the WDR1 does not enhance this interaction on the *DFR* promoter. In combination with Ros 1, Inc I gave similar increases in luciferase activity on the wild‐type *DFR* promoter but inclusion of the WDR1 protein slightly enhanced the induction of luciferase by Ros 1 and Inc I suggesting that Inc I and WDR1 interacted positively in the MBW complex to induce transcription from the *DFR* promoter.

Deletion of the sequences between −93 and −116 bp upstream of the initiating ATG removed the TATA‐1 box associated with TS1 (p2557). This deletion resulted in loss of induction of the *DFR* promoter except for the background response to Ros 1, suggesting that the MBW complex primarily regulates transcription of the *DFR* gene using TATA‐1 and TS1. The remaining transcriptional response to Ros 1 alone, presumably is mediated by TATA‐2 and TS2.

Assay of a mutant *DFR* promoter lacking 12 nucleotides immediately downstream of AC‐box 1 that included G‐box 1 known to be recognised by bHLH proteins, (p2534), abolished the induction of luciferase activity by both Del and Inc I (with or without the WDR1 protein) (Fig. 7b). These data indicated that both bHLH proteins, Del and Inc I, recognise and bind G‐box 1 to activate *DFR* gene expression. G‐box 1 is therefore likely bound by both bHLH proteins in the MBW complex and the Myb protein, Ros 1, likely binds to the AC‐box 1 in the wild‐type promoter especially when interacting with Del. Deletion of the *DFR* promoter involving loss of G‐box 1, AC‐box 1 and 200 nucleotides further upstream placed another G‐box (G‐box 2) −188 to −193 bp upstream of the initiating ATG codon of the *DFR* gene. Dual luciferase assays using this promoter mutation, which in *A. majus* results in almost complete loss of *DFR* expression (Almeida *et al*., 1989), did not restore responsiveness to Del nor to Inc I plus the WDR1 protein, over and above responsiveness to Ros 1 alone. This showed that the sequence context and proximity of the AC‐box and G‐box motifs relative to the basal transcriptional machinery assembled around the TATA box are important in defining the response of the target gene to the MBW complex.

To unravel the recognition of sequence motifs by the MBW complexes further, new mutations of the *DFR* promoter were created (p2551; p2565 and p2571). Removal of 12 bp downstream of G‐box 1 (loss of −154 to −165 bp), including AC‐box 2 (AACACC; −157 to −162; p2551) gave good induction of the *DFR* promoter in response to Ros 1 and Del, although inclusion of WDR1 in this assay inhibited luciferase activity to the levels achieved by Ros 1 alone (Fig. 7b). Interestingly, loss of AC‐box 2 eliminated responsiveness to Inc I and WDR1. We tested the effects of the MBW components on deletion of AC‐box 1 (−180 to −192; p2571; Fig. 7b). This caused large reductions in responsiveness of the *DFR* promoter to Del with Ros 1 and, by contrast to all other versions of the *DFR* promoter, the presence of WDR1 enhanced expression by the Ros 1–Del complex suggesting that Ros 1–Del binding to AC‐box 2 (in the absence of AC‐boz 1) and G‐box‐1 is enhanced by WDR1. Loss of AC‐box 1 eliminated the response to Inc I, although there was perhaps a small induction when Inc I was combined with WDR1. To confirm these results, we tested a promoter deletion that included both AC boxes and G‐box 1 (−154 to −192; p2565). This deletion completely eliminated responsiveness of the *DFR* promoter to the MBW complex (Fig. 7b), confirming that the MBW components work through the combined AC‐box 1, G‐box 1 and AC‐box 2 upstream activator sequence (UAS) and implying that there are structural and consequently functional differences in the way Ros 1 interacts with the bHLH‐1 and bHLH‐2 proteins on the *DFR* promoter. A model for how this might work is proposed in Fig. 7c.

**Fig. 8:**
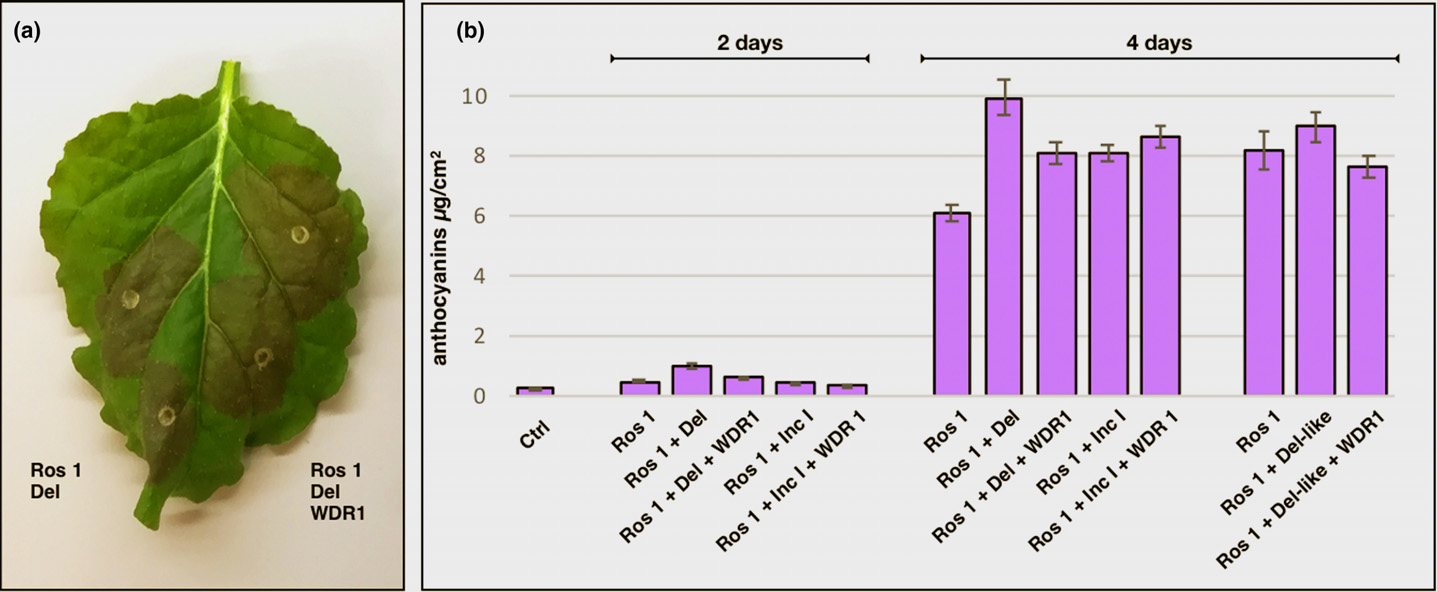
Anthocyanin production in leaves of *Nicotiana benthamiana* var NT in response to MBW components from *A. majus*. (a) Production of anthocyanin in *N. benthamiana* ‘NT’ leaves 4 d post inoculation. (b) Total anthocyanin production in response to MBW components of *A. majus* in *N. benthamiana* ‘NT’. Total anthocyanin production reflects the net effects of the MBW components on the activities of all anthocyanin biosynthetic enzymes and transporters. Error bars show SEMs

Of course, the effects of the MBW complex on anthocyanin production reflect the net responses of all anthocyanin biosynthetic genes and the relative changes in enzyme and transporter activity on anthocyanin accumulation. To investigate the net effect of the MBW components on the regulation of anthocyanin production in *N. benthamiana*, we tested different combinations of regulatory proteins for induction of anthocyanins in the leaves of *N. benthamiana* ‘Northern Territory’ which does not suffer from reported problems with induction of anthocyanins in leaves of the more commonly used laboratory strain (Bally *et al*., 2015; Thole *et al*., 2019). Anthocyanin levels were significantly induced by Ros 1 on its own (>25 fold) and these were further elevated by inclusion of a vector expressing Del in the transient assay (>45 fold) (Fig. 8). Inclusion of a vector expressing the WDR1 protein repressed the induction of anthocyanins by Ros 1 and Del significantly, suggesting that the combination of Ros 1 and Del is negatively impacted by inclusion of the WDR1 protein on anthocyanin production. Induction of anthocyanin production by the combination of Ros 1 and Inc I was not as great as for Ros 1 and Del, but was enhanced slightly by inclusion of the WDR1 protein suggesting that the WDR protein interacts positively with Inc I to promote net anthocyanin production by the MBW complex in *N. benthamiana*.

We also tested the functionality of *Del‐like* in inducing anthocyanin production in *N. benthamiana*. Although Del‐like did induce a small increase in anthocyanin produced in leaves, this was not significantly greater than Ros 1 alone. Inclusion of WDR1 in the inoculum reduced this small increase below levels for Ros 1 alone (Fig. 8). We concluded that the weak enhancement of anthocyanin biosynthesis by Del‐like in *N. benthamiana* suggested that it plays a negligible role in controlling anthocyanin biosynthesis directly in flowers of *A. majus*.

### The regulatory network controlling expression of the components of the MBW complex

In petunia, the bHLH‐1 protein (JAF13) forms an MBW complex that regulates the expression of the *An1* gene (*bHLH‐2*), enabling new MBW complexes to form with specific Myb proteins that target anthocyanin, proanthocyanidin and acidification pathways (Albert *et al*., 2014). While features of this hierarchical regulatory network have been reported in several plants (Baudry *et al*., 2006; Albert *et al*., 2014; Albert, 2015; Liu *et al*., 2014; Montefiori *et al*., 2015; Li *et al*., 2020), it is not known whether hierarchical regulation is universal.

**Fig. 9:**
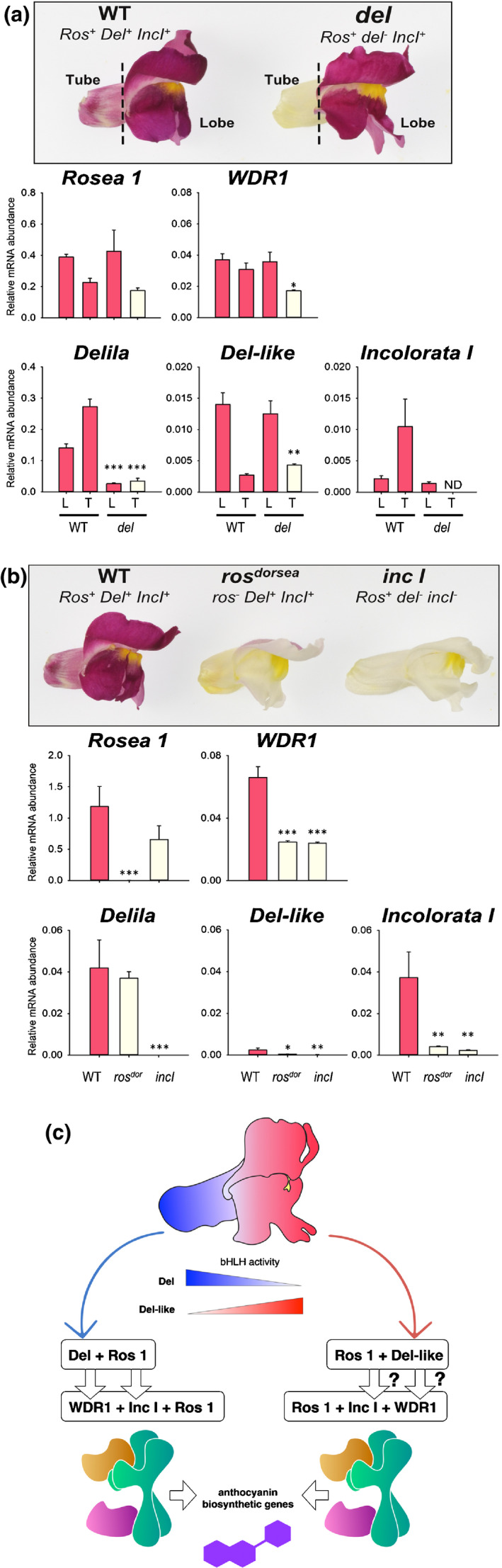
Bicolour patterning in the *delila* mutant arises from mis‐regulation of *Incolorata* I in flowers of *A. majus*. (a) Wild‐type (WT) and *delila* mutant flowers were analysed for *Rosea1*, *WDR1*, *Delila*, *Del‐like* and *Incolorata I* transcript levels by qRT‐PCR in tube (T) and lobe (L) tissue of WT and *delila* mutant petals. (b) Petals were analysed for *Rosea 1*, *WDR1*, *Delila*, *Del‐like* and *Incolorata I* transcript levels by qRT‐PCR. Bar colour indicates pigmentation status. Means ± SEM, *n* = 3 independent, biological replicates. Means that are significantly different (Student’s *t‐*test) from WT are indicated (*, *P* < 0.05; **, *P* < 0.01; ***, *P* < 0.001). (c) Model illustrating how bicolour patterning may result from the tube‐ and lobe‐centred activities of Del and Del‐like respectively regulating expression of *Inc I* and *WDR1* which, in turn, is necessary for full Inc I activity in the MBW complex.

Hierarchical regulation was investigated by analysing the expression of anthocyanin regulatory genes in petals (Fig. 9). *Ros 1* abundance was unaffected by the *del* mutation and transcripts were present in both tube and lobe tissues. *Inc I* transcripts were detected only in the lobes of *del* mutants but, interestingly, *Inc I* was expressed in both the tube and lobes of wild‐type petals, indicating that Del promotes *Inc I* expression in tubes. *Delila‐like* transcripts were detected in the lobes and tubes of both wild‐type and *del* mutants albeit at relatively low levels, suggesting that *Del‐like* is not regulated by the MBW complex. *WDR1* transcripts were reduced in the tube of *del* mutants compared with wild‐type, suggesting that its expression is controlled, in part, by the MBW complex.

Analysis of transcript levels in *ros^dor^* lines confirmed which regulatory genes were under MBW control. *Inc I* showed significantly lower levels of expression in *ros^dor^* compared with wild‐type flowers, confirming that it was under MBW control. Although *Del‐like* showed reduced expression in *ros^dor^*, the transcript levels detected were so low as to make the significance of the reduction ambiguous. *Del* was not impacted in its transcript levels in *ros^dor^* compared with wild‐type, confirming that *Del* is not regulated by the MBW complex. *Ros 1* expression in *inc I *:* del* showed no significant changes in transcript levels compared with wild‐type, confirming that *Ros 1* is not controlled by the MBW complex.

## Discussion

We have identified *Incolorata I* encoding a bHLH‐2 protein involved in regulating anthocyanin biosynthesis in flowers of *A. majus*. Inc I has partially overlapping functions with Del, a bHLH‐1 protein. Both regulate anthocyanin biosynthesis directly and their differential activity in flowers determines bicoloured patterning of petals in *del* mutants. This differential activity of the two transcription factors can also explain the weak, patterned pigmentation of mutations of the *Pallida* gene encoding DFR, an enzyme essential for anthocyanin biosynthesis (Coen *et al*., 1986; Almeida *et al*., 1989). However, Inc I and Del are not completely functionally redundant; Del positively regulates *Inc I* (and *WDR1*) expression in flower tubes, and it is the Del‐independent expression of *Inc I* that gives bicoloured patterning of flowers. It is possible that Del‐independent, lobe‐specific expression of *Inc I* is dependent on Del‐like, which is expressed more highly in lobes than in tubes. It is unlikely that Del‐like regulates anthocyanin biosynthesis directly because *inc I *:* del* homozygotes completely lack anthocyanins in their flowers, or in any other parts of the plant. In addition, the Myb transcription factor Ros 1 showed no significant promotion of anthocyanin production in combination with Del‐like (Fig. 8) in *N. benthamiana*. Although Del‐like does not appear to activate the expression of anthocyanin biosynthetic genes substantially it might activate *Inc I* expression specifically in lobes.

Activity of Ros 1 and Del in inducing anthocyanin biosynthesis is not enhanced by the presence of the WDR1 protein and may, in fact, be inhibited by it. Activity of Ros 1 with Inc I is enhanced by the presence of WDR1, meaning that Inc I may act in an MBW complex, but that the most effective complex of Ros1 and Del may lack the WDR1 protein. Competition between Myb and WDR proteins in forming complexes with bHLH‐1 proteins has been reported by Pesch *et al*. (2015) in trichome formation in Arabidopsis, with the conclusion that this competition results in either an MB complex or a BW complex that regulate expression of different genes. In an extensive analysis of different subgroup IIIf bHLH proteins, Zhang *et al*. (2019) demonstrated that only bHLH‐1 proteins show competitive complex formation with Myb and WDRs whereas bHLH‐2 proteins always show enhanced complex formation with Myb and WDR proteins. This same difference between the bHLH proteins was shown by net anthocyanin biosynthesis in *N. benthamiana*, reflecting the integrated output of the MBW complex in leaves and implying that this difference also impacted expression of other genes involved in anthocyanin accumulation (Fig. 8).

Del and Inc I bind to G‐Box 1 in the *DFR* promoter of *A. majus*. Both positioning and sequence context of this G‐box are likely to be important for the binding and activity of Inc I and Del, as introduction of a G‐box displaced upstream in a different sequence context did not rescue the induction of gene expression driven by Del or Inc I from the *DFR* promoter lacking G‐box 1. The residual tube pigmentation observed in the equivalent mutants of *A. majus* (*pal‐*32 and *pal*‐33; Almeida *et al*., 1989) is likely to be driven by Del association with Ros 1 even where G‐box 1 has been deleted, so preventing DNA binding by bHLH transcription factors. However, binding by the bHLH protein to the target gene DNA may not be essential for inducing low level anthocyanin production in *A. majus*, (Fig. S8a). This ability of Del to interact with Ros 1 and promote anthocyanin production without DNA binding has been reported in tobacco (Applehagen *et al*., 2018), and for the B bHLH protein in regulation of the *bz1* promoter in maize (Goff *et al*., 1992). Interestingly, the residual pigmentation observed in *pal*‐32 and *pal‐*33 is restricted predominantly to the flower tube (Fig. S8a; Coen *et al*., 1986; Almeida *et al*., 1989) suggesting that, although DNA binding may not be necessary for Del participation in the MBW complex on the *DFR* promoter, DNA binding is probably essential for Inc I‐WDR1 association with the MBW complex and *DFR* expression in the lobes in *A. majus* (Fig. S8b). This residual contribution of Del to induction of *DFR* gene expression is considerably lower than activation mediated by its binding to G‐box 1, and might vary from target gene to target gene as several of these promoter sequences in *A. majus* lack G‐box or even E‐box motifs (for example *F3H*: Martin *et al*., 1991) that are bound by bHLH proteins.

Bicolour patterning in flowers of *A. majus* results from *Inc I* being controlled by Del in tubes, reinforced by Del controlling *WDR1* expression, which contributes positively to the activity of the Inc I‐containing MBW complex. In lobes there is Del‐independent expression of *Inc I* but this expression is probably dependent on an MB complex, possibly involving Del‐like (which is more highly expressed in lobes than tubes) interacting with Ros 1. Del‐like might have hierarchical activity on anthocyanin production through regulating *Inc I*, similar to the role of JAF13 in petunia (Albert *et al*., 2014) and tobacco (Montefiori *et al*., 2015). These findings reinforce the view that bHLH‐1 and bHLH‐2 proteins differ in their activity, even when their target pathways overlap, and that pigmentation patterning is established through hierarchical activities of transcription factors. The identification of transcription factors and mechanisms responsible for anthocyanin bicolouration in *Antirrhinum* provides a model for future studies to elucidate how the regulators of tissue and organ identity are linked to domain‐specific pigmentation patterns.

## Author contributions

CM, PP, NWA, SMAM and KES cloned and characterised the *Incolorata I* gene and its mutant alleles, SMAM characterised the *del^23^* mutant allele. CM and KES undertook RNA analysis using RNA gel blots and NWA analysed gene expression by qRT‐PCR. CNW performed biolistic complementation assays and KES cloned *WDR1*. EB performed dual luciferase assays in *N. benthamiana*, made all mutations of the *DFR* promoter for these assays and prepared Figs 7, 8, 9. NWA, KMD and CM drafted the manuscript before all authors contributed to its improvement and agreed on its final content. NWA and EB contributed equally to this work.

## Supporting information


**Dataset S1** Amino acid alignment of bHLH proteins.
**Fig. S1** Differences in *mutabilis* phenotypes between glasshouse and field‐grown plants of *A. majus*.
**Fig. S2** Lack of complementation between *inc I1* and *inc I2* of *A. majus*.
**Fig. S3** RNA gel blot showing lack of *Inc I* transcript in flower lobes of the *mutabilis* mutant of *A. majus*.
**Fig. S4** Sectors caused by excision of a transposable element (Tam 2) from the *Del* gene in *inc I2 *: *delrec* plants of *A. majus*.
**Fig. S5** Phenotype of *del23* allele of *A. majus*.
**Fig. S6** Molecular analysis of *del23* of *A. majus*.
**Fig. S7** Impaired expression of anthocyanin biosynthetic genes in flowers of the *inc I2* mutant of *A. majus*.
**Fig. S8** Phenotypes of *Pallida* mutants of *A. majus* with deletions in their UAS controlling *DFR* expression caused by imprecise transposon excision and described by Almeida *et al* (1989).
**Table S1** Primers used in this study.
Please note: Wiley Blackwell are not responsible for the content or functionality of any Supporting Information supplied by the authors. Any queries (other than missing material) should be directed to the *New Phytologist* Central Office.
