# Supplementary material for: Discrete bHLH transcription factors play functionally overlapping roles in pigmentation patterning in flowers of Antirrhinum majus
Source: New Phytol. 2021 Jan 12;231(2):849–63. doi: 10.1111/nph.17142 (PMC8248400; doi:10.1111/nph.17142)
Supplement: Supplementary file 1 — Dataset S1 Amino acid alignment of bHLH proteins. Fig. S1 Differences in mutabilis phenotypes between glasshouse and field‐grown plants of A. majus. Fig. S2 Lack of complementation between inc I1 and inc I2 of A. majus. Fig. S3 RNA gel blot showing lack of Inc I transcript in flower lobes of the mutabilis mutant of A. majus. Fig. S4 Sectors caused by excision of a transposable element (Tam 2) from the Del gene in inc I2 : delrec plants of A. majus. Fig. S5 Phenotype of del23 allele of A. majus. Fig. S6 Molecular analysis of del23 of A. majus. Fig. S7 Impaired expression of anthocyanin biosynthetic genes in flowers of the inc I2 mutant of A. majus. Fig. S8 Phenotypes of Pallida mutants of A. majus with deletions in their UAS controlling DFR expression caused by imprecise transposon excision and described by Almeida et al (1989). Table S1 Primers used in this study. Please note: Wiley Blackwell are not responsible for the content or functionality of any Supporting Information supplied by the authors. Any queries (other than missing material) should be directed to the New Phytologist Central Office. [file NPH-231-849-s001.pdf]

New Phytologist Supporting Information

Article title: Discrete bHLH transcription factors play functionally overlapping roles in pigmentation patterning in flowers of Antirrhinum majus

Authors: Nick W. Albert, Eugenio Butelli, Sarah M. A. Moss, Paolo Piazza, Chethi N. Waite, Kathy E. Schwinn, Kevin M. Davies and Cathie Martin

Article acceptance date: 30 November 2020

Contents:

Supporting Figure S1: Differences in *mutabilis* phenotypes between greenhouse and field-grown plants of *A.majus*

Supporting Figure S2 Lack of complementation between *inc I*^{1} and *inc I*^{2} of *A.majus*

Supporting Figure S3: RNA gel blot showing lack of *Inc I* transcript in flower lobes of the *mutabilis* mutant of *A.majus*

Supporting Figure S4 Sectors caused by excision of a transposable element (Tam 2) from the *Del* gene in *inc I*^{2}*: del*^{rec} plants of *A.majus*

Supporting Figure S5 Phenotype of *del*^{23} allele of *A.majus*

Supporting Figure S6: Molecular analysis of *del*^{23} of *A.majus*

Supporting Figure S7: Impaired expression of anthocyanin biosynthetic genes in flowers of the *inc I*^{2} mutant of *A.majus*

Supporting Figure S8: Phenotypes of *pallida* mutants of *A.majus* with deletions in their UAS controlling *DFR* expression caused by imprecise transposon excision and described by Almeida *et al* (1989)

Supporting Dataset S1: Amino acid alignment of bHLH proteins

Supporting Table S1: Primers used in this study

Supporting references

Supporting Figure S1: Differences in *mutabilis* phenotypes of *A. majus* between greenhouse and field-grown plants.

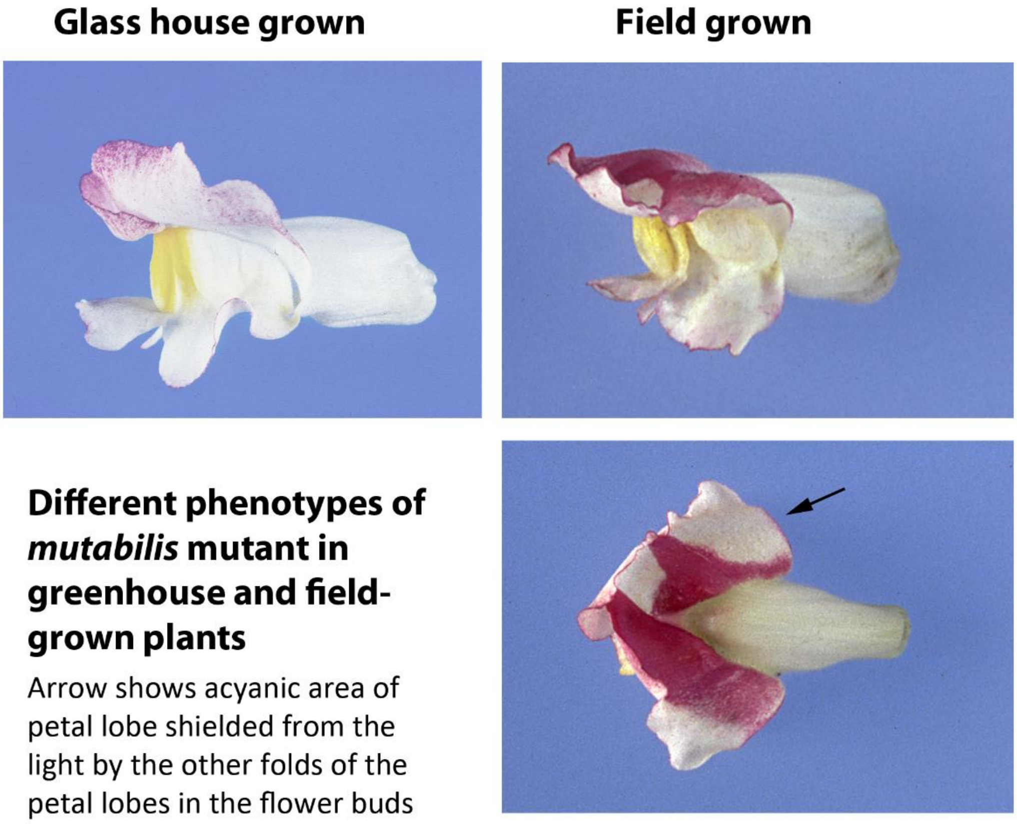

Supporting Figure S2:

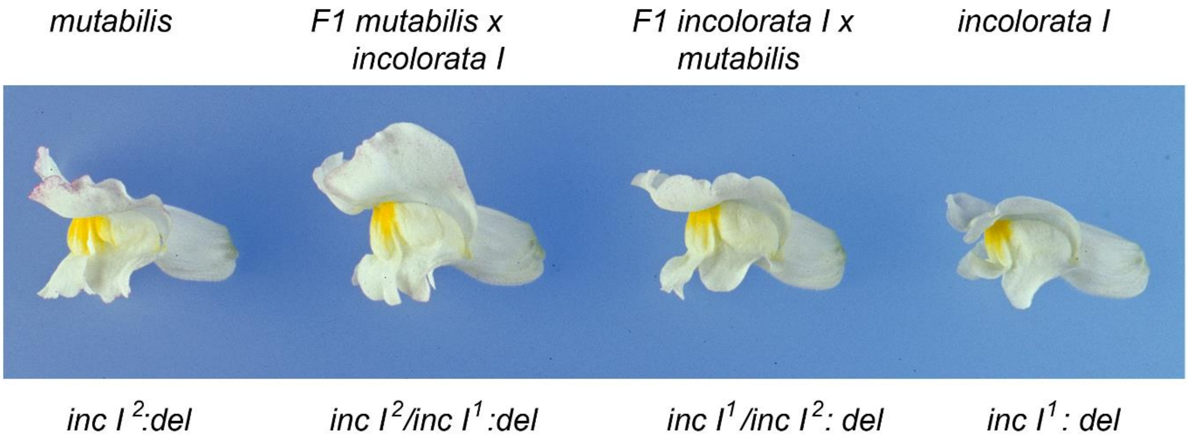

Supporting Figure S2: Lack of complementation between *inc I^1* and *inc I^2 in A. majus*
The phenotypes of the *mutabilis* and *incolorata I* mutants are shown with the phenotypes of two of their F_1 progeny**.**

Supporting Figure S3:

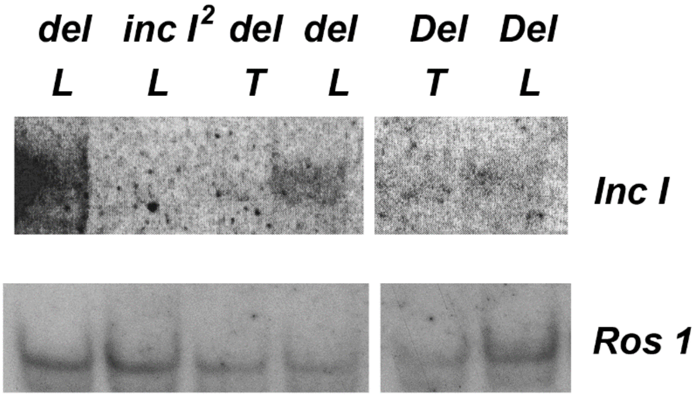

Supporting Figure S3: RNA gel blot showing lack of *Inc I* transcript in flowers of the *mutabilis* mutant *of A. majus*

Total RNA from flower lobes (L) from the *inc I*[2] mutant lack *Inc I* transcript compared to total RNA from lobes of *del* mutants. No *Inc I* transcript was detected in flower tubes (T) of the *del* mutant. *Inc I* transcript was detected in lobes of wild type (*Del*) flowers and also faintly in tube tissue from wildtype flowers. Transcript levels of *Ros 1* in the same RNA preparations are shown below as loading controls. The *Inc I* probe was pJAM1494, the *Ros 1* probe was pJAM1450 (Schwinn *et al*., 2006).

Supporting Figure S4:

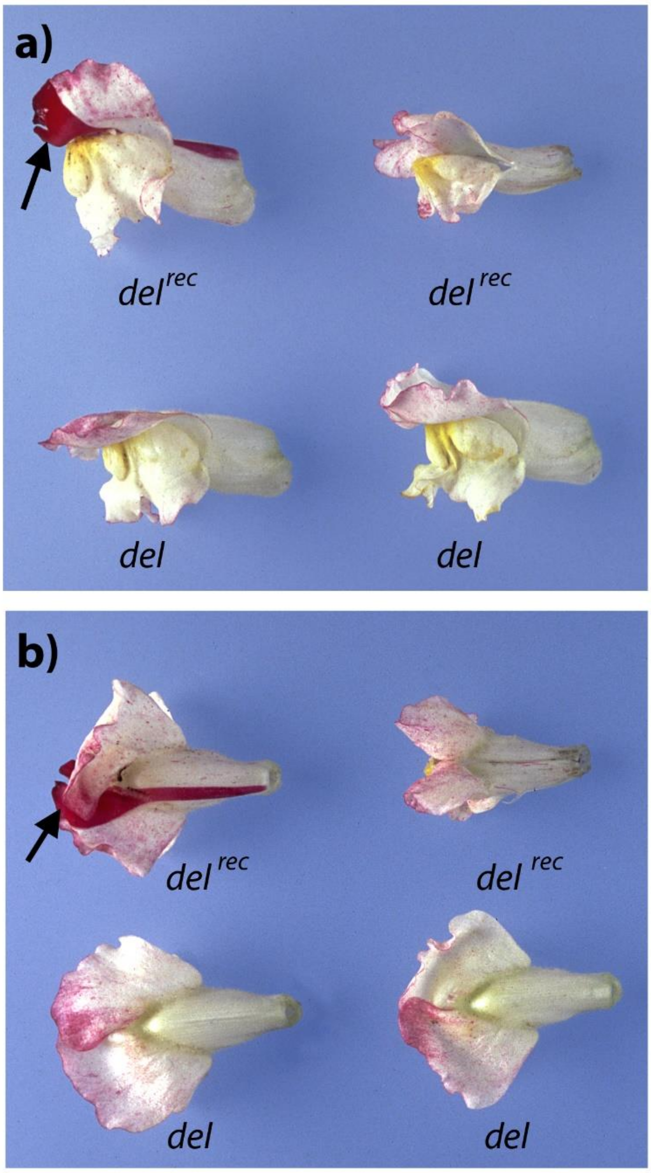

Supporting Figure S4: Sectors caused by excision of the transposable element (Tam 2) from the *Del* gene in *inc I*^{2}*: del*^{rec} *A. majus* plants showing that Del activity can complement Inc I activity in flower lobes. Arrows point to a *Del*$^{+,}$ full red revertant sector that includes both lobe and tube tissue in an *inc I*^{2}*/inc I*^{2}: *del*^{rec} */del*^{rec} mutant line **a)** shows side view of flowers **b)** shows dorsal view of same flowers.

Supporting Figure S5:

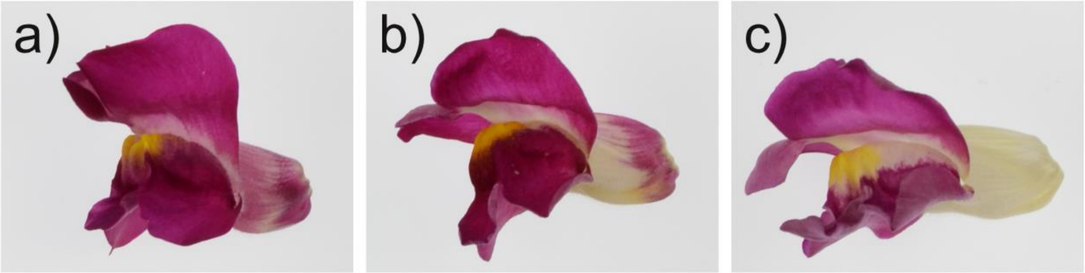

Supporting Figure S5: Phenotype of *del*^{23} allele of *A. majus*
a) shows wild type *Del* phenotype **b)** shows *del*^{23} phenotype **c)** shows *del* loss-of-function phenotype

Supporting Figure S6:

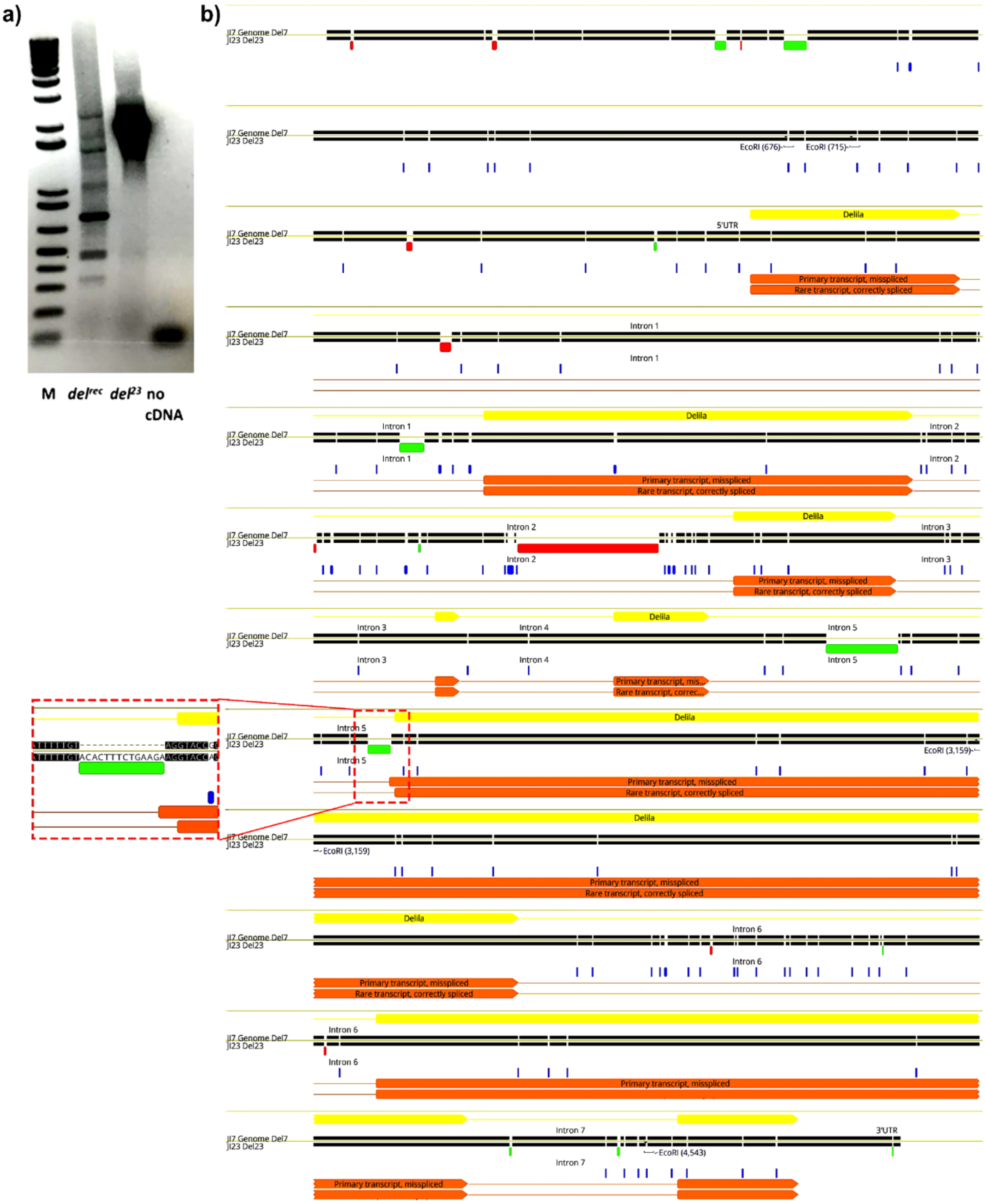

Supporting Figure S6: Molecular analysis of *del*^{23} of *A. majus*

a) qRT-PCR amplification of *Del* transcript in flowers of *del*^{rec} and *del*^{23} lines. **b)** Nucleotide sequence alignment of the reference genome WT JI:7 (upper sequence) and JI:23 (lower sequence). Annotations of sequence variants relative to the JI:7 reference genome are as follows: single nucleotide polymorphisms are indicated in blue, insertions in green, deletions in red below the aligned genomic sequences. The predominant transcript amplified from JI:23 flowers spliced into an insertion within intron five (see enlarged sequence in dashed-red box), inserting one additional amino acid (indicated by upper transcript shown scarlet bar). Rarely, the *del*^{23} transcript spliced correctly (indicated by lower transcript shown as scarlet bar).

Supporting Figure S7:

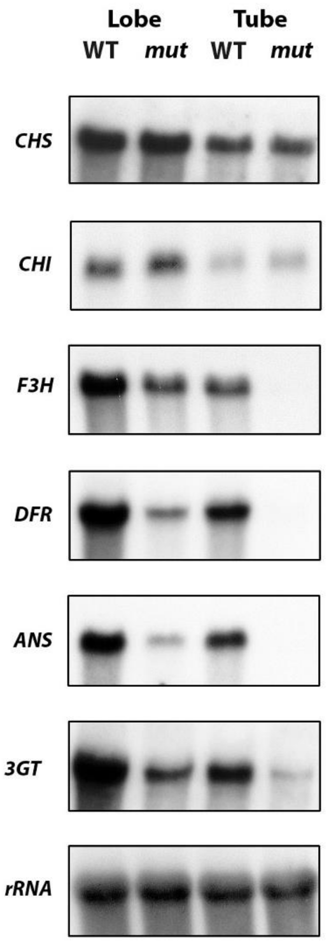

Supporting Figure S7: **Impaired expression of anthocyanin biosynthetic genes in flowers of the *inc I*^{2} mutant of *A. majus***

RNA gel blots of total RNA (25 µg) were hybridised with ^{32}P dCTP-labelled probes for the anthocyanin biosynthetic genes *CHS, CHI, F3H, DFR, ANS* and *3GT* or the 26S rRNA (loading control). RNA gel blots showed reduced transcript abundance for the anthocyanin biosynthetic genes *F3H, DFR, ANS* and *3GT* in the lobes of *inc I*^{2} mutants compared with wild-type. This confirmed that the weak pigmentation in *inc I*^{2} is because of impaired activation of the anthocyanin biosynthetic genes, and that *Inc I* encodes a regulator of anthocyanin biosynthesis.

Supporting Figure S8

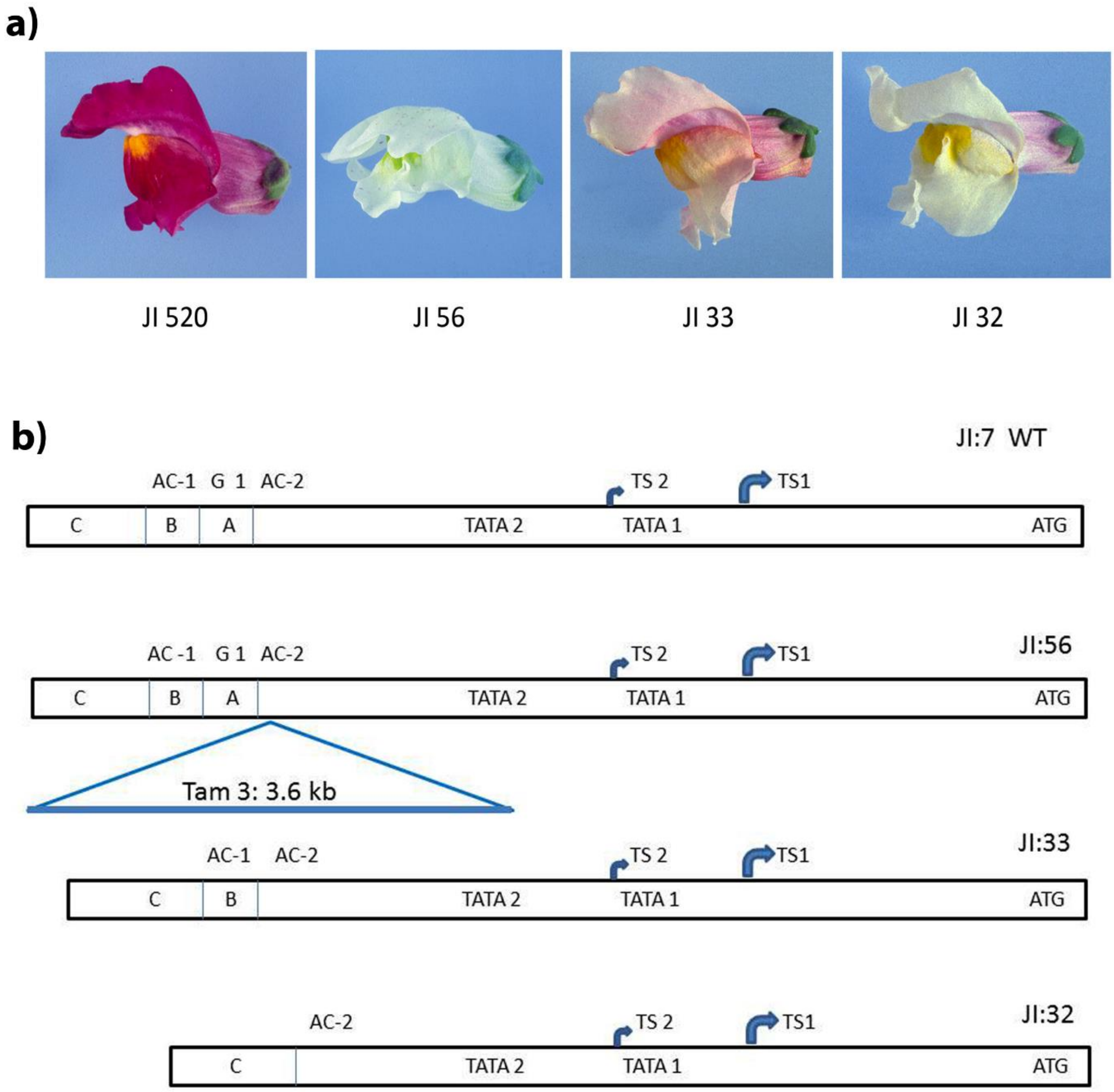

Supporting Figure S8: Phenotypes of *pallida* mutants of *A. majus* with deletions in their UAS controlling *DFR* expression caused by imprecise transposon excision and described by Almeida *et al* (1989)

a) Phenotypes of *pallida* alleles affected in the structure of the *DFR* promoter in *A. majus*.

b) Diagram of the UAS in *DFR* promoter in WT (JI:520) and mutant alleles of the *Pallida* locus encoding *DFR* in *A. majus*. The sequence of the UAS interacting with the MBW complex defined in this study (AC-box 1, G-box 1 and AC-box 2) are shown for each allele as well as the regions of the DFR promoter, boxes A, B and C, defined as important for regulation by Almeida *et al*., (1989). TS1 = transcriptional start site 1, TATA 1 = TATA box associated with TS1, TS2 = transcriptional start site 2 and TATA 2 = TATA box associated with TS2 in *DFR* promoter of *A. majus* (Coen *et al.*, 1986).

Supporting Dataset S1: Amino acid alignment of bHLH proteins. MUSCLE alignment of bHLH proteins used for Maximum likelihood phylogenetic tree in Figure 2c.

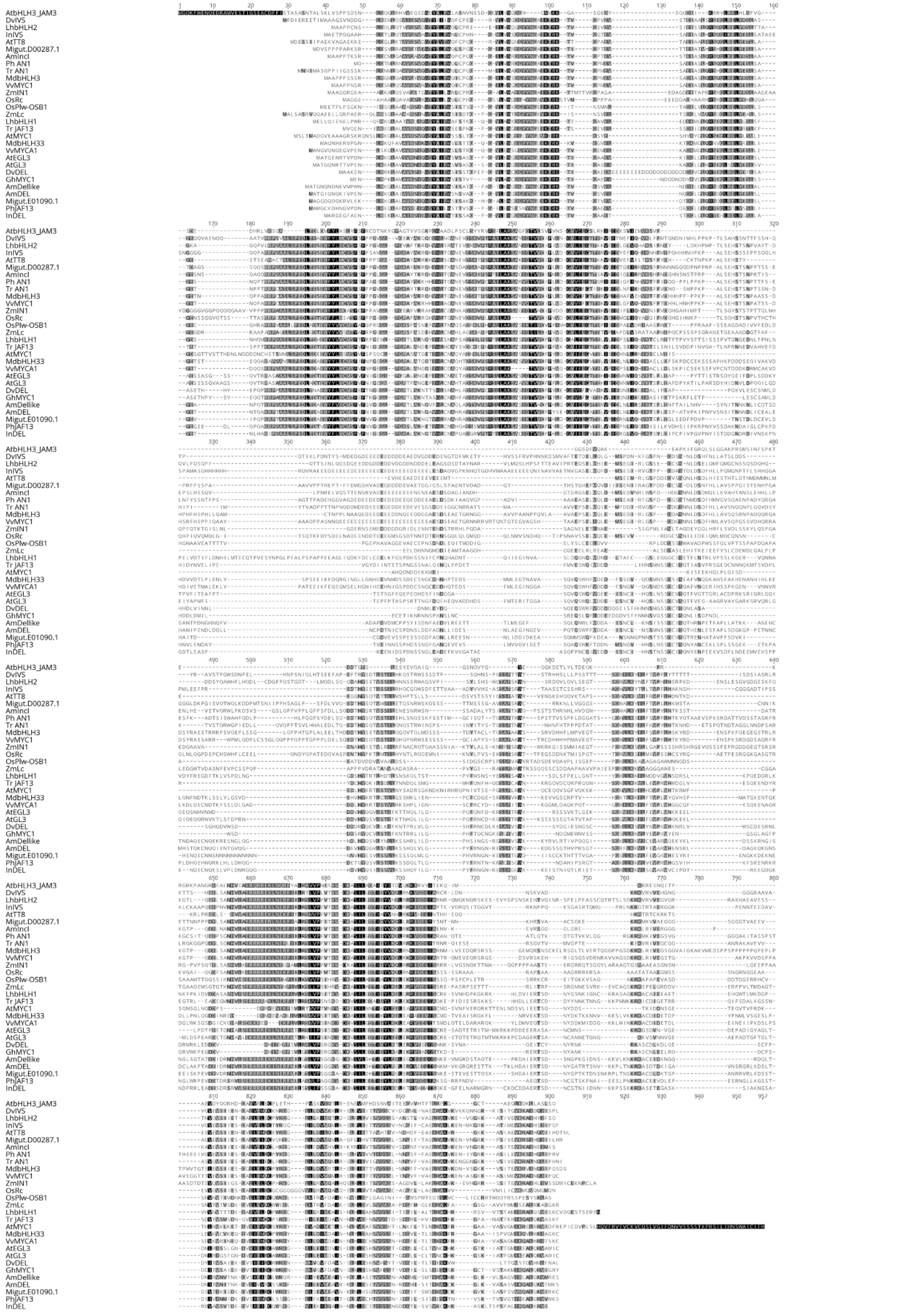

Supporting Table S1: Primers used in this study			
**Number**	**Primer**	**Sequence**	**Application**
NA587	*Incolorata I* Am02g53780	CTAAGAATTCAAGTTGCATCAGGC	Sequencing
NA588	*Incolorata I* Am02g53780	AGCCTTAGTTTGGTTCCCAGG	Sequencing
NA589	*Incolorata I* Am02g53780	GTGAGATTCACAATAGCTTTGGC	Sequencing
NA590	*Incolorata I* Am02g53780	CAAGTCAGAAAATTAGGTGGCG	Sequencing
NA591	*Incolorata I* Am02g53780	TGAATGTTCTCTGCACATGCC	Sequencing
NA592	*Incolorata I* Am02g53780	GCGCGAAACCACATGAAGG	Sequencing
SM119	*Incolorata I* Am02g53780	GAGAAGTACTAAAATGGCTGCAC	Sequencing
SM120	*Incolorata I* Am02g53780	CTTGGCTCCGCACCTAGTG	Sequencing
SM121	*Incolorata I* Am02g53780	CTAGTGTGGCGTGATGGATATTAC	Sequencing
SM122	*Incolorata I* Am02g53780	GCTGTTTGATTCTCCGGCAG	Sequencing
SM123	*Incolorata I* Am02g53780	GTTCGCATAACACTAGTAACCG	Sequencing
SM124	*Incolorata I* Am02g53780	CCTTAGCAAGAATTGTCCTGGA	Sequencing
SM125	*Incolorata I* Am02g53780	CATCCGAGTCCGACTCAGC	Sequencing
SM126	*Incolorata I* Am02g53780	GTGCTTCCGAATCGTGAACT	Sequencing
SM127	*Incolorata I* Am02g53780	GAGAATAGGTGATTATACGGGACA	Sequencing
SM128	*Incolorata I* Am02g53780	CCATGGACAAGTCAGTAGGCC	Sequencing
SM129	*Incolorata I* Am02g53780	CCCGAATTTTGACTAGCCAAATAC	Sequencing
SM130	*Incolorata I* Am02g53780	CTCTCTCTCAGCCCTGAAAGC	Sequencing
SM131	*Incolorata I* Am02g53780	CACTGAACAGCTTGAACCGC	Sequencing
SM132	*Incolorata I* Am02g53780	AGGCTGTAAGTCCACTGAACAG	Sequencing
SM133	*Incolorata I* Am02g53780	GTTAATCACTCGGTTCATTTCAATTAG	Sequencing
SM134	*Incolorata I* Am02g53780	TGGCATGGTTGAAGCAAGAC	Sequencing
SM135	*Incolorata I* Am02g53780	CTTACTTGTATTCCAGCACTCTTG	Sequencing
SM136	*Incolorata I* Am02g53780	CACACAAAAGGGGTGGAGGAG	Sequencing
SM137	*Incolorata I* Am02g53780	GGTGAAGGAGAATGTGAATGGTAG	Sequencing
SM138	*Incolorata I* Am02g53780	TTAGACTGAGAAGCCGGAGC	Sequencing
NA560	*Delila (del^{23} allele)*	CACCaaaATGGCTACTGGTGTCCAAAAC	Cloning/ Sequencing
NA561	*Delila (del^{23} allele)*	TCAAGACTTCATAATAACTTTCTGAAGAG	Cloning/ Sequencing
SM112	*Delila* Am02g33340	GGATTCAAGAATGGCTACTGGT	Sequencing
SM113	*Delila* Am02g33340	AGCCCTGCAAATTACTTAGCAA	Sequencing
SM114	*Delila* Am02g33340	GGTGATGGGTTCTACAATGGAG	Sequencing
SM115	*Delila* Am02g33340	GATTCAATTCGACAGATTGTACAGT	Sequencing
SM116	*Delila* Am02g33340	GTGCGGACACCAAAGTTTTC	Sequencing
SM117	*Delila* Am02g33340	GCTTTTGAATTGTCCAGACACG	Sequencing
SM118	*Delila* Am02g33340	GAAACCACGTCTTGTCAGAGAG	Sequencing
NA556	*Del-like* Am02g28470	CACCAAAATGGCAACTGGAAACCAAAATGATA ATG	Cloning/ Sequencing
NA559	*Del-like* Am02g28470	TCAAGACTCCCTCATAACCTTCTGC	Cloning/ Sequencing
NA557	*Del-like* Am02g28470	CTACCGCAATTCTCAACCATGTC	Sequencing

NA558	*Del-like* Am02g28470	CGGCCACAAACTGATTATGACC	Sequencing
NA568	*Del-like* Am02g28470	GGTTGTGTAGCTGAGATGGAC	Sequencing
NA569	*Del-like* Am02g28470	GACCTAGTTGAGACACTCGAC	Sequencing
NA570	*Del-like* Am02g28470	TGGGCAGATCAGGGAGAC	Sequencing
NA571	*Del-like* Am02g28470	ATCGATTTGTGTGTGTGGAAC	Sequencing
K112	*WDR1*	ATHGAYACIACITGYACIATHTGGGA	3'RACE
K113	*WDR1*	TGGAAYAARCARGAYYTIMGITAYATGGC	3'RACE
(dT)17-adaptor	cDNA synthesis for 3'RACE Frohman et al., 1988	GACTCGAGTCGACATCGATTTTTTTTTTTTTTTTT	3'RACE
adaptor	Adapter primer for 3'RACE Frohman et al., 1988	GACTCGAGCGACATCGAT	3'RACE
K127	*WDR1*	GCACCAAACATCGACATGGGAACAATC	Gene walking
K128	*WDR1*	AGCAACCGTCGGCAGTTCCCACAGCAA	Gene walking
K133	*WDR1*	tgcaGGTACCAATGGACATTTCAACCCAC	Cloning cDNA
K134	*WDR1*	tcgaTCTAGAGAGAGCTAAAGCCGTCGAC	Cloning cDNA
K564	*Am EF1a* Am01g17900	GACTGCCACACCTCCCACATTG	qPCR
K565	*Am EF1a* Am01g17900	TCACCATACCAGCGTCACCATTC	qPCR
K566	*Am Cyclophilin* Am05g43400	CCAGGGCGGCGATTTCACC	qPCR
K567	*Am Cyclophilin* Am05g43400	GCGTTCGCCATGGACAGGATTC	qPCR
K488	*Nivea*/CHS Am04g40840	TGCTGCGTATGGCGAAGGACT	qPCR
K489	*Nivea*/CHS Am04g40840	CCGCGGTAACGATCTGGAAAA	qPCR
K461	*Pallida*/DFR Am06g29640	CACATCAATGGACAAGAGAATGC	qPCR
K462	*Pallida*/DFR Am06g29640	GCCATCAGTATGATCGTTTGC	qPCR
NA621	*Candi*/ANS Am02g33790	GGTTGAGGAGAAGGAGAAGC	qPCR
NA622	*Candi*/ANS Am02g33790	CCACTCCAGTTGACCACTAG	qPCR
NA623	3-*O*-UFGT Am02g24600	TGAGAGTGTTATGGTCGTGG	qPCR
NA624	3-*O*-UFGT Am02g24600	CTCCTGAACTCCCTTATCACC	qPCR
NA625	CHI Am02g23710	CTGCGATCACCCAAATTCAG	qPCR
NA626	CHI Am02g23710	ACTTGATGAACTTCCCCTGG	qPCR
NA631	*Inc II*/F3H Am05g03330	TACTTTGCTGCTCCAGGATC	qPCR
NA632	*Inc II*/F3H Am05g03330	GAGTTCACAACTGCTTGGTG	qPCR
K562	*Rosea1* Am06g36450	AGGATGGGGAATTAGGAAACCTA	qPCR
K563	*Rosea1* Am06g36450	CTCTCCATAACATCAGTAATCTC	qPCR
K297	*WDR1* Am01g20780	TGGGCGCCGCACAGTTG	qPCR
K299	*WDR1* Am01g20780	GCTAAAGCCGTCGACTACCTCACA	qPCR
SM13	*Incolorata I* Am02g53780	CCTCGTGCCGTTTGTTACTA	qPCR
SM14	*Incolorata I* Am02g53780	CTCGAGCTCCTCAATCTTCTTC	qPCR
SM15	*Delila* Am02g33340	ATTCTTGCATCCCTAGTCCCATCC	qPCR
SM16	D*elila* Am02g33340	CCCCCGGCCCTTTACCATTTT	qPCR
NA562	*Del-like* Am02g28470	TGTGGAGGCAAAGAGTGATTTA	qPCR
NA563	*Del-like* Am02g28470	CCCTTCCACTTGGCTCTTATAG	qPCR
EB-665	*Pallida*/DFR promoter Am06g29640	GTTATCACGTGCCTCGCGA	Deletion for p2551 and

			p2552
EB-666	*Pallida*/DFR promoter Am06g29640	TTTAGGAAGGTGATAGTATTACGTACT	Deletion for p2551 and p2565
EB-668	*Pallida*/DFR promoter Am06g29640	GTATAAACTATGCCACTTCTATGACA	Deletion for p2552
EB-672	*Pallida*/DFR promoter Am06g29640	TTTATATAGTACGTAATACTATCACCTTC	Deletion for p2557
EB-674	*Pallida*/DFR promoter Am06g29640	AAGCTTATTCAAAAATCGTATAGAAATTCA	Deletion for p2557
EB-697	*Pallida*/DFR promoter Am06g29640	GTCTTGGAAAGTTAGGAGAATTTCGT	Deletion for p2565 and p2571
EB-719	*Pallida*/DFR promoter	AGGCACGTGATAACCCTACCA	Deletion for p2571

Supplementary references:

Almeida J, Carpenter R, Robbins TP, Martin C, Coen ES. 1989. Genetic interactions underlying flower color patterns in *Antirrhinum majus*. *Genes & Development* **3**(11): 1758-1767.

Coen ES, Carpenter R, Martin C. 1986. Transposable elements generate novel spatial patterns of gene-expression in *Antirrhinum majus*. *Cell* **47**(2): 285-296.

Schwinn K, Venail J, Shang Y, Mackay S, Alm V, Butelli E, Oyama R, Bailey P, Davies K, Martin C. 2006. A small family of MYB-regulatory genes controls floral pigmentation intensity and patterning in the genus *Antirrhinum*. *Plant Cell* **18**(4): 831-851.
